# OSU-6: A Highly Efficient, Metal-Free, Heterogeneous Catalyst for the Click Synthesis of 5-Benzyl and 5-Aryl-1*H*-tetrazoles

**DOI:** 10.3390/molecules201219881

**Published:** 2015-12-19

**Authors:** Baskar Nammalwar, Nagendra Prasad Muddala, Rajasekar Pitchimani, Richard A. Bunce

**Affiliations:** Department of Chemistry, Oklahoma State University, Stillwater, OK 74078-3071, USA; baskar@okstate.edu (B.N.); npiict@gmail.com (N.P.M.); raja2765@yahoo.com (R.P.)

**Keywords:** 5-benzyl and 5-aryl-1*H*-tetrazoles, carboxylic acid bioisosteres, click 1,3-dipolar addition, heterogeneous catalysis, recyclable catalyst

## Abstract

OSU-6, an MCM-41 type hexagonal mesoporous silica with mild Brönsted acid properties, has been used as an efficient, metal-free, heterogeneous catalyst for the click synthesis of 5-benzyl and 5-aryl-1*H*-tetrazoles from nitriles in DMF at 90 °C. This catalyst offers advantages including ease of operation, milder conditions, high yields, and reusability. Studies are presented that demonstrate the robust nature of the catalyst under the optimized reaction conditions. OSU-6 promotes the 1,3-dipolar addition of azides to nitriles without significant degradation or clogging of the nanoporous structure. The catalyst can be reused up to five times without a significant reduction in yield, and it does not require treatment with acid between reactions.

## 1. Introduction

Tetrazoles are versatile heterocyclic systems, which have attracted considerable interest in diverse applications ranging from pharmaceuticals [1,2,3] and agrochemicals [4] to photographic compounds [5], explosives [6], new materials [7,8,9], and ligands in coordination compounds [10,11]. In biological studies, tetrazoles play a critical role as pharmacophores and also as metabolic surrogates for carboxylic acids in various therapeutic agents to treat cancer, AIDS, bacterial infections, hypertension, convulsions, and allergies [12,13]. Currently, the commercial antihypertensives Losartan and Valsartan [14] as well as an experimental 2-arylcarbapenem antibiotic [15] all incorporate a tetrazole ring within their structures. Tetrazoles have been synthesized primarily by the reaction of azides with nitriles in polar aprotic media. This process has been promoted by various metal-based agents, including aluminum chloride, aluminum bisulfate, cadmium chloride, copper-, zinc- and iron-based salts, copper delafossite nanoparticles, palladium complexes, silver benzoate, metal-based triflates, tungstates, zinc-copper alloys, and zinc sulfate nanospheres [16,17]. Additionally, other heterogeneous catalysts, including CoY zeolites [18,19], SiO_2_–H_2_SO_4_ [20], Amberlyst-15 [21], and cuttlebone [22], as well as soluble additives NH_4_OAc and NH_4_Cl [23,24] have also been used to facilitate this reaction. We therefore wish to report a new catalyst which performs this conversion under metal-free conditions, at moderate temperatures, and with superior conversion rates, stability toward traces of water, and recyclability.

## 2. Results and Discussion

OSU-6, an MCM-41 type hexagonal mesoporous silica [25], has recently proven useful as a mildly acidic, reusable catalyst for several transformations in our laboratory, affording products in excellent yields with minimal purification requirements [26,27,28]. Encouraged by these results, we sought to explore OSU-6 as a catalyst for the click synthesis of tetrazoles from azide and nitriles [29,30]. In order to gauge the feasibility of this process, the reaction of benzyl cyanide (**1a**, 1 equiv.) with sodium azide (1.2 equiv.) to generate 5-benzyl-1*H*-tetrazole (**2a**) was chosen as a model reaction. Various solvents were evaluated using 15 wt % of the catalyst (relative to **1a**) under varying temperature conditions (Table 1). Our optimization study determined that the reaction performed in DMF solvent at 90 °C using 15 wt % of OSU-6, afforded the highest yield (94%) of the tetrazole product. More or less catalyst did not improve the yields, and lower temperatures led to inefficient conversions. In general, polar aprotic media gave superior results since they solubilized both reacting partners at temperatures ≥90 °C. Polar protic and nonpolar media, on the other hand, led to unsatisfactory outcomes. Solventless conditions afforded reasonable conversions for the model reaction, but were not practical for solid nitriles, and the requisite higher temperatures led to greater impurity profiles. Earlier syntheses [23,31] generally utilized reaction temperatures of 120–150 °C for this reaction, and thus, the conditions employed in this work are somewhat milder.

**Table 1:** Reaction optimization. 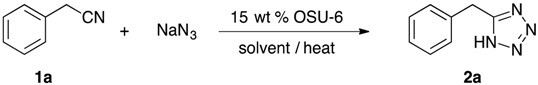

Entry	Solvent	OSU-6 (wt %)	Temperature (^o^C)	Time (h)	Isolated Yield (%)
1	EtOH	15	90	12	trace
2	CH_3_CN	15	90	24	18
3	dioxane	15	95	18	trace
4	THF	15	70	18	10
5	solventless	15	120	6	76
6	DMSO	15	140	6	84
7	DMF	50	120	6	88
8	DMF	25	120	6	87
9	DMF	15	120	6	90
10 ^a^	DMF	15	90	4	94
11	DMF	15	75	6	10
12	DMF	10	90	6	73
13	DMF	0	120	12	trace

^a^ Optimized conditions.

Based on our preliminary findings, we now report our investigation of this catalyst for the synthesis of 5-benzyl- and 5-aryl-1*H*-tetrazoles using a click approach. The optimized conditions (15 wt % OSU-6, DMF, 90 °C, 4–12 h) proved general for promoting the conversion of benzyl and aryl nitriles to tetrazoles. In addition to the parent systems, substrate derivatives bearing methyl, methoxy, fluoro, chloro, nitro, and 3-butenyl moieties were evaluated, and the results are summarized in Table 2. All of these groups survived the reaction conditions and gave high yields of products, regardless of their electronic character or position on the ring, thus demonstrating the general applicability of OSU-6 in this transformation. Furthermore, in the conversion of 4-(3-butenyl)benzonitrile (**1p**) to tetrazole **2p**, the reaction showed excellent chemoselectivity for the nitrile over the terminal alkene. Finally, attempts to react aliphatic nitriles lacking aromatic substitution gave incomplete conversion to the target tetrazoles and unacceptable impurity levels under our conditions.

**Table 2:** Synthesis of tetrazoles. 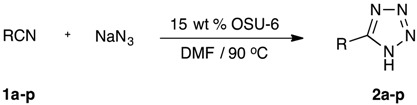

Substrate	R	Product	Time (h)	Isolated Yield (%)
**1a**	C_6_H_5_CH_2_	**2a**	4	94
**1b**	4-CH_3_C_6_H_4_CH_2_	**2b**	5	87
**1c**	4-CH_3_OC_6_H_4_CH_2_	**2c**	4	92
**1q**	4-ClC_6_H_4_CH_2_	**2d**	5	90
**1e**	4-FC_6_H_4_CH_2_	**2e**	5	86
**1f**	C_6_H_5_	**2f**	8	89
**1g**	4-CH_3_C_6_H_4_	**2g**	8	90
**1h**	2-CH_3_C_6_H_4_	**2h**	6	94
**1i**	4-CH_3_OC_6_H_4_	**2i**	6	92
**1j**	4-NO_2_C_6_H_4_	**2j**	12	84
**1k**	3-NO_2_C_6_H_4_	**2k**	12	80
**1l**	4-ClC_6_H_4_	**2l**	8	91
**1m**	4-FC_6_H_4_	**2m**	8	87
**1n**	(C_6_H_5_)_2_CH	**2n**	12	94
**1o**	4-CH_3_(CH_2_)_6_C_6_H_4_	**2o**	4	87
**1p**	4-CH_2_=CH(CH_2_)_2_C_6_H_4_	**2p**	4	95

**Scheme 1:**
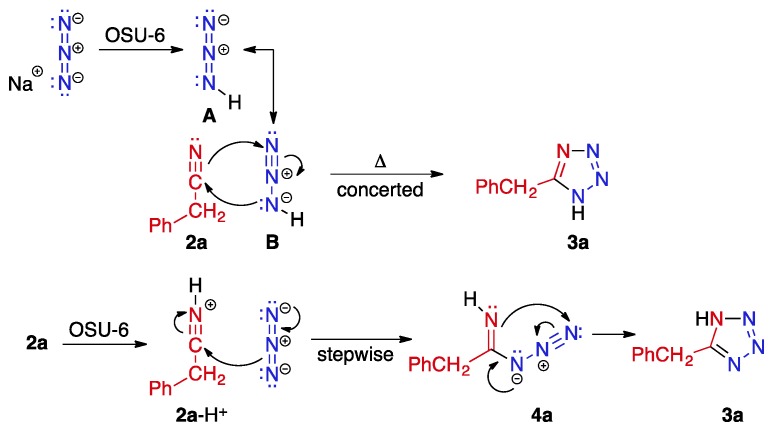
Plausible mechanisms for the reaction of azide with nitrile **2a** in the presence of OSU-6 to form tetrazole **3a**.

The 1,3-dipolar addition of azide to nitriles has the potential to proceed via a concerted or stepwise mechanism. In either event, OSU-6 would serve as a mild proton source to convert azide to hydrazoic acid or to activate the nitrile function by protonation (Scheme 1). Hydrazoic acid has two major resonance contributors, **A** and **B**. Of these, contributor **B** illustrates the concerted reaction best, undergoing a smooth six-electron cyclization with nitrile **2a** to form tetrazole **3a**. It is also possible that OSU-6 protonates the nitrile to some degree, which would activate this group toward attack by azide in a stepwise process. Many earlier papers, both with [14,32,33] and without [12,19,23] metal catalysts, have presented stepwise mechanisms for this transformation. In our experiments, however, no intermediates were observed by thin layer chromatography during the course of the reaction. Additionally, the minimal substitution in the reactants provided no stereochemical evidence to illuminate the concerted or stepwise nature of the process.

**Figure 1:**
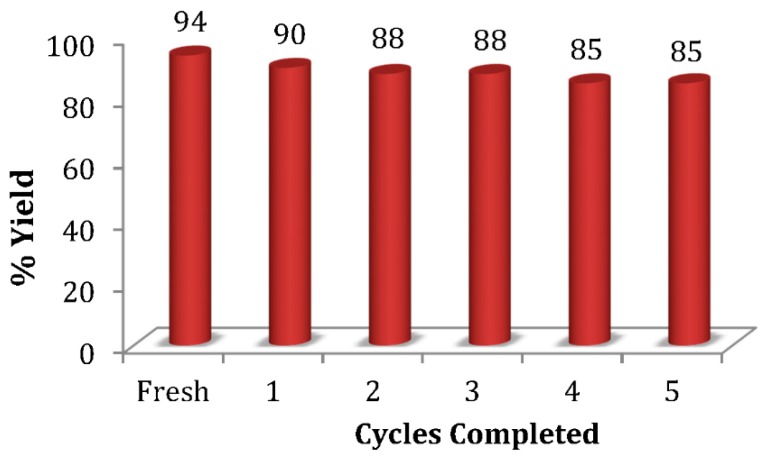
Recyclability of OSU-6.

One of the most attractive features of OSU-6 in our previous studies [26,27,28] was its recyclability, and thus, the catalyst was evaluated for this possibility. The acidity of this silicic material apparently derives from aging in 2 M HCl for two weeks during its preparation [34]. In the current application, OSU-6 was reused up to five times without significant loss of activity (Figure 1) and required only minimal reconditioning after each cycle. This reconditioning involved filtering the catalyst, washing with 1:1 EtOH:H_2_O, and drying under high vacuum at 80 °C for 2 h. Employing this protocol, OSU-6 retained its proton donor properties without the need for acid treatment between reactions.

**Figure 2:**
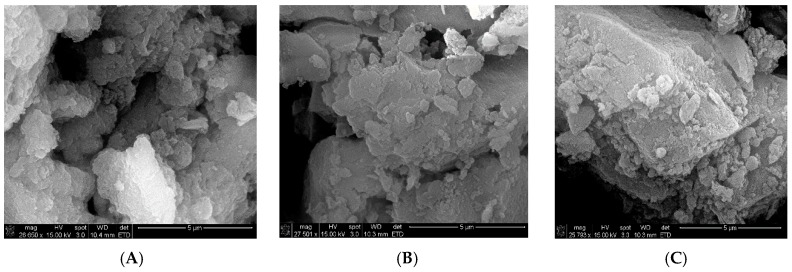
SEM images of OSU-6: (**A**) Fresh; (**B**) After three cycles; and (**C**) After five cycles.

In order to understand the high catalytic activity of OSU-6, we monitored changes to the catalyst surface over five cycles of the reaction using scanning electron microscopy (SEM). A series of SEM images at ~26,000× magnification (Figure 2) show 100 µm^2^ areas on the catalyst surface before and after use. Comparison of the surface topography of fresh OSU-6 (**A**) with material recovered after the third (**B**) and fifth (**C**) reactions revealed that while some roughening occurred due to the loss of water, the exposed features of the catalyst remained largely unchanged. Overall, the catalyst morphology showed only minor observable degradation after five iterations.

**Figure 3:**
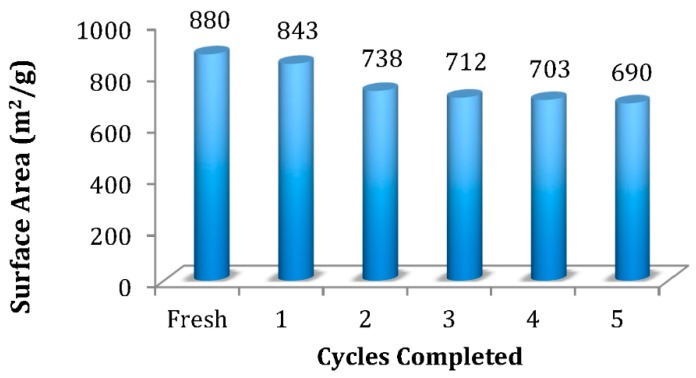
BET measurements of the surface area of OSU-6.

The change in catalyst structure during multiple reactions was further investigated for one charge of catalyst using a Brunauer-Emmett-Teller (BET) surface area determination. The results revealed that fresh OSU-6 had a total surface area of 880 m^2^/g. Measurements after each reaction cycle showed a gradual decrease in surface area, with a total loss of only 21.6% to 690 m^2^/g after five cycles (Figure 3). Thus, the catalyst suffered only minimal damage and was able to retain its pore size and volume throughout the five-reaction sequence. These observations verified the structural stability of OSU-6 toward conditions requiring extended exposure to a polar aprotic solvent at 90 °C.

**Figure 4:**
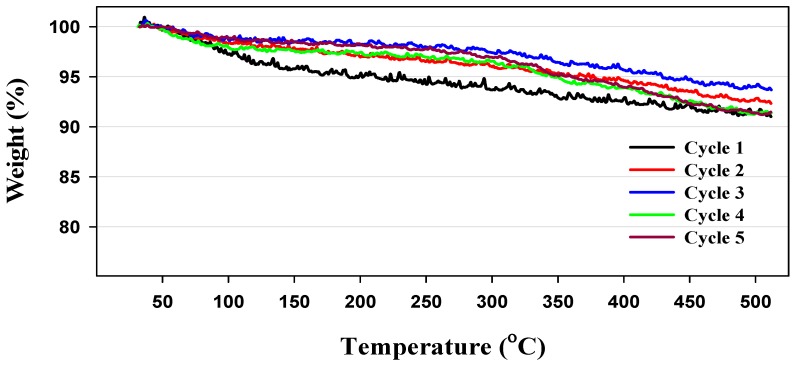
Thermogravimetric analysis (TGA) of recovered OSU-6.

In a further study of the behavior of OSU-6 over an extended reaction series, thermogravimetric analysis (TGA) was used to assess the extent of clogging in the catalyst pores by reagents and products. The results revealed a weight loss of 2%–4% below 150 °C, corresponding to the loss of adsorbed water. Upon further heating, relatively little additional weight loss (≤6%) was noted from the reused OSU-6 samples between 150–500 °C, indicating that the catalyst pores experienced no significant clogging during the reaction (Figure 4). Lastly, though not shown in the Figure, OSU-6 did not breakdown until the temperature reached *ca.* 800 °C, confirming the robust character of the material.

Finally, to more fully elucidate the role of OSU-6 in the reaction, two separate experiments were performed on the model conversion of benzyl cyanide (**1a**, 1.0 mmol) and sodium azide (1.2 mmol) to **2a** under the optimized conditions (15 wt % of OSU-6, DMF, 90 °C, 4 h). In the first run, the reaction was heated in the presence of OSU-6 for a period of 2 h, after which the catalyst was removed by filtration and the filtrate was heated for a second 2-h period. This procedure gave roughly 68% of **2a** during the first 2 h, but only an additional 5% of product during the subsequent 2-h period. The sequence was then reversed, heating the reaction in the absence of OSU-6 for the initial 2-h period, followed by introduction of catalyst and heating for another 2 h. This procedure yielded only ~10% of **2a** after the first 2 h, but an additional 76% during the second 2-h period. These results clearly established that OSU-6 was essential in promoting the click addition process to form tetrazoles.

## 3. Experimental Section

### 3.1. General Information

The OSU-6 catalyst can be purchased from XploSafe, LLC (Product No. 9001, Stillwater, OK, USA; www.xplosafe.com). All reactions were run under dry N_2_. Reactions were monitored by thin layer chromatography (TLC) on silica gel GF plates (Analtech No. 21521, Newark, DE, USA). Column chromatography, when necessary, was performed using silica gel (Davisil^®^ grade 62, 60–200 mesh) mixed with UV-active phosphor (Sorbent Technologies, No. UV-05, Norcross, GA, USA); band elution was monitored using a hand held UV lamp. The following instrumentation was used: Mp determinations: Laboratory Devices Mel-temp apparatus (Cambridge, MA, USA); FT-IR spectra: Varian Scimitar FTS 800 spectrophotometer (Randolph, MA, USA); ^1^H- (400 MHz) and ^13^C-NMR (100 MHz) spectra: Bruker Avance 400 system (Billerica, MA, USA); HRMS: Thermo LTQ-Orbitrap XL system (Waltham, MA, USA); BET surface areas: Quantachrome Autosorb 1 instrument (Boynton Beach, FL, USA); SEM images: Hitachi S4800 system (Shaumburg, IL, USA); TGA measurements: Mettler-Toledo TGA/DSC 1 instrument (Columbus, OH, USA).

### 3.2. Representative Procedure for the Preparation of 5-Benzyl and 5-Aryl-1H-tetrazoles

*5-Benzyl-1H-tetrazole* (**2a**). To a DMF solution of benzyl cyanide (**1a**, 100 mg, 0.85 mmol) and NaN_3_ (67 mg, 1.02 mmol, 1.2 eq) was added OSU-6 (15 mg, 15 wt% relative to **1a**). The reaction mixture was heated at 90 °C (oil bath temperature 95–100 °C) for 4 h at which time TLC indicated the reaction was complete. The crude reaction mixture was filtered to remove the catalyst, and the filtrate was added to water and extracted with EtOAc (3 × 15 mL). The combined extracts were washed with H_2_O (3 × 15 mL) and saturated aq. NaCl (1 × 15 mL), dried (MgSO_4_), filtered, and concentrated under vacuum to give **2a** (129 mg, 94%) as a white solid, mp 121–122 °C (lit. [31] mp 123–124 °C). IR: 1603, 1549, 1532, 1494, 1457, 1073, 733, 695 cm^−1^; ^1^H-NMR (DMSO-*d*_6_): δ 16.1 (br s, 1H), 7.34 (t, *J* = 7.4 Hz, 2H), 7.27 (d, *J* = 7.4 Hz, 3H), 4.29 (s, 2H); ^13^C-NMR (DMSO-*d*_6_) δ 155.7, 136.4, 129.1, 128.8, 127.5, 29.4; HRMS (ESI): *m*/*z* Calcd for C_8_H_8_N_4_: 161.0827 [M + H]; Found: 161.0831. Other tetrazoles (below) were prepared in the same fashion by heating for the times indicated in Table 2.

*5-(4-Methylbenzyl)-1H-tetrazole* (**2b**): Yield: 115 mg (87%) as a white solid, mp 151–152 °C (lit. [31] mp 153–154 °C); IR: 1635, 1540, 1496, 1448, 1382, 869 cm^−1^; ^1^H-NMR (DMSO-*d*_6_): δ 16.1 (br s, 1H), 7.15 (apparent s, 4H), 4.23 (s, 2H), 2.27 (s, 3H); ^13^C-NMR (DMSO-*d*_6_) δ 154.6, 135.5, 132.2, 128.6, 127.9, 27.9, 20.0; HRMS (ESI): *m*/*z* Calcd for C_9_H_10_N_4_: 175.0984 [M + H]; Found: 175.0982.

*5-(4-Methoxybenzyl)-1H-tetrazole* (**2c**): Yield: 119 mg (92%) as an off-white solid, mp 160–162 °C (lit. [35] mp 162–164 °C); IR: 2834, 1613, 1514, 1246, 836 cm^−1^; ^1^H-NMR (DMSO-*d*_6_): δ 16.1 (br s, 1H), 7.19 (d, *J* = 8.2 Hz, 2H), 6.90 (d, *J* = 8.2 Hz, 2H), 4.21 (s, 2H), 3.72 (s, 3H); ^13^C-NMR (DMSO-*d*_6_) δ 157.7, 155.0, 129.2, 127.1, 113.7, 54.5, 27.4; HRMS (ESI): *m*/*z* Calcd for C_9_H_10_N_4_O: 191.0933 [M + H]; Found: 191.0938.

*5-(4-Chlorobenzyl)-1H-tetrazole* (**2d**): Yield: 115 mg (90%) as a white solid, mp 155–156 °C; IR: 1641, 1586, 1536, 1493, 1409, 827, 764 cm^−1^; ^1^H-NMR (DMSO-*d*_6_): δ 16.1 (br s, 1H), 7.41 (d, *J* = 8.1 Hz, 2H), 7.31 (d, *J* = 8.1 Hz, 2H), 4.30 (s, 2H); ^13^C-NMR (DMSO-*d*_6_) δ 154.5, 134.3, 131.1, 130.0, 128.0, 27.6; HRMS (ESI): *m*/*z* Calcd for C_8_H_7_N_4_Cl: 195.0438, 197.0408 (*ca.* 3:1) [M + H]; Found: 195.0442, 197.0411 (*ca.* 3:1). Anal. Calcd for C_8_H_7_N_4_Cl: C, 49.37; H, 3.63; N, 28.79. Found: C, 49.46; H, 3.55; N, 28.89.

*5-(4-Fluorobenzyl)-1H-tetrazole* (**2e**): Yield: 113 mg (86%) as a light yellow solid, mp 154–155 °C; IR: 1601, 1582, 1508, 1413, 1223, 828, 767 cm^−1^; ^1^H-NMR (DMSO-*d*_6_): δ 16.1 (br s, 1H). 7.33 (m, 2H), 7.18 (t, *J* = 8.7 Hz, 2H), 4.30 (s, 2H); ^13^C-NMR (DMSO-*d*_6_) δ 160.7 (d, *J* = 241 Hz), 154.7, 131.5 (d, *J* = 4 Hz), 130.1 (d, *J* = 8.1 Hz), 114.9 (d, *J* = 22.2 Hz), 27.5; HRMS (ESI): *m*/*z* Calcd for C_8_H_7_N_4_F: 179.0733 [M + H]; Found: 179.0737. Anal. Calcd for C_8_H_7_N_4_F: C, 53.93; H, 3.96; N, 31.45. Found: C, 54.02; H, 3.92; N, 31.37.

*5-Phenyl-1H-tetrazole* (**2f**): Yield: 126 mg (89%) as an off-white solid, mp 215–216 °C [lit. [36] mp 216 °C (dec)]; IR: 1608, 1563, 1485, 1465, 1409, 1162, 746, 703 cm^−1^; ^1^H-NMR (DMSO-*d*_6_): δ 8.09–8.03 (m, 2H), 7.66–7.57 (m, 3H), tetrazole NH off-scale; ^13^C-NMR (DMSO-*d*_6_) δ 130.7, 128.8, 126.4, 123.5; HRMS (ESI): *m*/*z* Calcd for C_7_H_6_N_4_: 147.0671 [M + H]; Found: 147.0668.

*5-(4-Methylphenyl)-1H-tetrazole* (**2g**): Yield: 122 mg (90%) as a tan solid, mp 249–250 °C (lit. [31] mp 247.5–247.7 °C); IR: 1612, 1569, 1505, 1369, 822, 742 cm^−1^; ^1^H-NMR (DMSO-*d*_6_): δ 7.94 (d, *J* = 7.8 Hz, 2H), 7.42 (d, *J* = 7.8 Hz, 2H), 2.40 (s, 3H), tetrazole NH off-scale; ^13^C-NMR (DMSO-*d*_6_) δ 154.5, 140.6, 129.7, 126.3, 120.9, 20.5; HRMS (ESI): *m*/*z* Calcd for C_8_H_8_N_4_: 161.0827 [M + H]; Found: 161.0829.

*5-(2-Methylphenyl)-1H-tetrazole* (**2h**): Yield: 128 mg (94%) as an off-white solid, mp 152–153 °C (lit. [31] mp 153.2–153.8 °C); IR: 1608, 1564, 1465, 1387, 782, 744 cm^−1^; ^1^H-NMR (DMSO-*d*_6_): δ 7.70 (d, *J* = 7.6 Hz, 1H), 7.54–7.36 (m, 3H), 2.51 (d, *J* = 2.0 Hz, 3H), tetrazole NH off-scale; ^13^C-NMR (DMSO-*d*_6_) δ 154.7, 136.5, 130.7, 130.1, 128.8, 125.7, 123.3, 19.9; HRMS (ESI): *m*/*z* Calcd for C_8_H_8_N_4_: 161.0827 [M + H]; Found: 161.0831.

*5-(4-Methoxyphenyl)-1H-tetrazole* (**2i**): Yield: 122 mg (92%) as a white solid, mp 230–232 °C (lit. [35] mp 232–233 °C); IR: 2859, 1608, 1249, 828, 749 cm^−1^; ^1^H-NMR (DMSO-*d*_6_): δ 8.01 (d, *J* = 8.4 Hz, 2H), 7.18 (d, *J* = 8.4 Hz, 2H), 3.86 (s, 3H), tetrazole NH off-scale; ^13^C-NMR (DMSO-*d*_6_) δ 160.9, 154.1, 128.0, 115.7, 114.3, 54.9; HRMS (ESI): *m*/*z* Calcd for C_8_H_8_N_4_O: 177.0776 [M + H]; Found: 177.0773.

*5-(4-Nitrophenyl)-1H-tetrazole* (**2j**): Yield: 109 mg (84%) as a yellow solid, mp 219–221 °C (lit. [35] mp 218–220 °C); IR: 1603, 1551, 1512, 1337, 1318, 1293, 861, 728 cm^−1^; ^1^H-NMR (DMSO-*d*_6_): δ 8.46 (d, *J*= 8.4 Hz, 2H), 8.32 (d, *J* = 8.4 Hz, 2H), tetrazole H off-scale; ^13^C-NMR (DMSO-*d*_6_) δ 154.9, 148.2, 130.0, 127.6, 124.0; HRMS (ESI): *m*/*z* Calcd for C_7_H_5_N_5_O_2_: 192.0522 [M + H]; Found: 192.0525.

*5-(3-Nitrophenyl)-1H-tetrazole* (**2k**): Yield: 103 mg (80%) as a tan solid, mp 153–155 °C (lit. [31] mp 144.7–145.6 °C); IR: 1625, 1525, 1348, 1250, 872, 823, 712 cm^−1^; ^1^H-NMR (DMSO-*d*_6_): δ 8.84 (s, 1H), 8.49 (d, *J* = 7.7 Hz, 1H), 8.44 (d, *J* = 8.3 Hz, 1H), 7.92 (t, *J* = 8.0 Hz, 1H), tetrazole NH off-scale; ^13^C-NMR (DMSO-*d*_6_) δ 155.5, 148.7, 133.5, 131.7, 126.6, 126.1, 122.0; HRMS (ESI): *m*/*z* Calcd for C_7_H_5_N_5_O_2_: 192.0522 [M + H]; Found: 192.0528.

*5-(4-Chlorophenyl)-1H-tetrazole* (**2l**): Yield: 119 mg (91%) as a yellow solid, mp 259–260 °C (lit. [33] mp 262 °C); IR: 1635, 1535, 1492, 1419, 886, 755 cm^−1^; ^1^H-NMR (DMSO-*d*_6_): δ 8.07 (d, *J* = 8.2 Hz, 2H), 7.71 (d, *J* = 8.2 Hz, 2H), tetrazole NH off-scale; ^13^C-NMR (DMSO-*d*_6_) δ 155.4, 136.4, 130.0, 129.2, 123.7; HRMS (ESI): *m*/*z* Calcd for C_7_H_5_N_4_Cl: 181.0281, 183.0252 (*ca.* 3:1) [M + H]; Found: 181.0283, 183.0255 (*ca.* 3:1).

*5-(4-Fluorophenyl)-1H-tetrazole* (**2m**): Yield: 117 mg (87%) as an off-white solid, mp 203–205 °C; IR: 1606, 1499, 1444, 1241, 839, 749 cm^−1^; ^1^H-NMR (DMSO-*d*_6_): δ 8.14–8.04 (m, 2H), 7.48 (t, *J* = 8.6 Hz, 2H), tetrazole NH off-scale; ^13^C-NMR (DMSO-*d*_6_) δ 163.1 (d, *J* = 247. Hz), 154.2 (br), 128.9 (d, *J* = 9.1 Hz), 120.3, 116.0 (d, *J* = 21.5 Hz); HRMS (ESI): *m*/*z* Calcd for C_7_H_5_N_4_F: 165.0577 [M + H]; Found: 165.0581. Anal. Calcd for C_7_H_5_N_4_F: C, 51.22; H, 3.07; N, 34.13. Found: C, 51.36; H, 3.11; N, 34.29.

*5-(Diphenylmethyl)-1H-tetrazole* (**2n**): Yield, 109 mg, (94%) as a white solid, mp 161–163 °C (lit. [31] mp 164.2–165.2 °C); IR: 1564, 1494, 1452, 767, 718 cm^−1^; ^1^H-NMR (DMSO-*d*_6_): δ 7.40–7.25 (m, 10H), 5.97 (s, 1H), tetrazole NH off-scale; ^13^C-NMR (DMSO-*d*_6_) δ 139.5, 128.1, 127.8, 126.6, 45.1; HRMS (ESI): *m*/*z* Calcd for C_20_H_16_N_4_: 313.1453 [M + H]; Found: 313.1461.

*5-(4-Heptylphenyl)-1H-tetrazole* (**2o**): Yield: 105 mg (87%) as a white solid, mp 184–186 °C; IR: 1615, 1504, 1438, 1352, 844, 751, 722 cm^−1^; ^1^H-NMR (DMSO-*d*_6_): δ 7.94 (d, *J* = 7.8 Hz, 2H), 7.43 (d, *J* = 7.9 Hz, 2H), 2.66 (t, *J* = 7.7 Hz, 2H), 1.60 (quintet, *J* = 7.0 Hz, 2H), 1.30 (m, 4H), 1.25 (m, 3H), 0.85 (t, *J* = 6.6 Hz, 3H), tetrazole NH off-scale; ^13^C-NMR (DMSO-*d*_6_) δ 145.4, 145.3, 128.7, 126.3, 120.9, 34.4, 30.6, 30.1, 28.0, 27.9, 21.5, 13.3; HRMS (ESI): *m*/*z* Calcd for C_14_H_20_N_4_: 245.1766 [M + H]; Found: 245.1771. Anal. Calcd for C_14_H_20_N_4_: C, 68.82; H, 8.25; N, 22.93. Found: C, 68.87; H, 8.19; N, 22.85.

*5-[4-(3-Butenyl)phenyl]-1H-tetrazole* (**2p**): Yield: 121 mg (95%) as a yellow solid, mp 189–191 °C; IR: 1641, 1618, 1509, 1478, 998, 918, 844, 743 cm^−1^; ^1^H-NMR (DMSO-*d*_6_): δ 7.95 (d, *J* = 7.8 Hz, 2H), 7.45 (d, *J* = 7.9 Hz, 2H), 5.84 (ddt, *J* = 17.1, 10.2, 6.5 Hz, 1H), 5.05 (d, *J* = 17.1 Hz, 2H), 4.98 (d, *J* = 10.2 Hz, 1H), 2.77 (t, *J* = 7.6 Hz, 2H), 2.38 (q, *J* = 7.0 Hz, 2H), tetrazole NH off-scale; ^13^C-NMR (DMSO-*d*_6_) δ 145.1, 137.7, 130.0, 129.4, 126.9, 121.7, 115.5, 34.5, 34.3; HRMS (ESI): *m*/*z* Calcd for C_11_H_12_N_4_: 201.1140 [M + H]; Found: 201.1137. Anal. Calcd for C_11_H_12_N_4_: C, 65.98; H, 6.04; N, 27.98. Found: C, 65.90; H, 6.11; N, 28.09.

## 4. Conclusions

In summary, we have successfully used OSU-6 as an efficient, metal-free, heterogeneous catalyst for the high-yield click synthesis of 5-benzyl- and 5-aryl-1*H*-tetrazoles from nitriles in DMF at 90 °C. This MCM-41 type hexagonal mesoporous silica permits the synthesis of these targets using a simple procedure, under mild conditions, and can be readily recycled. Studies are presented which demonstrate the robust properties of the catalyst under the optimized reaction conditions. The catalyst promotes the 1,3-dipolar addition without significant surface erosion or clogging of the nanoporous structure. The catalyst can be reused up to five times without a significant reduction in yield, and it does not require treatment with acid between reactions.

## Supplementary Materials

Electronic Supplementary Information (ESI) available: Copies of the ^1^H and ^13^C-NMR spectra for each tetrazole prepared, see http://www.mdpi.com/1420-3049/20/12/19881/s1.
