# Supplementary material for: OSU-6: A Highly Efficient, Metal-Free, Heterogeneous Catalyst for the Click Synthesis of 5-Benzyl and 5-Aryl-1H-tetrazoles
Source: Molecules. 2015 Dec 19;20(12):22757–66. doi: 10.3390/molecules201219881 (PMC6332204; doi:10.3390/molecules201219881)

molecules

MDPI

Supplementary Materials: OSU-6: A Highly Efficient, Metal-Free, Heterogeneous Catalyst for the Click Synthesis of 5-Benzyl and 5-Aryl-1*H*-tetrazoles

Baskar Nammalwar, Nagendra Prasad Muddala, Rajasekar Pitchimani and Richard A. Bunce

5-Benzyl-1H-tetrazole (**2a**)

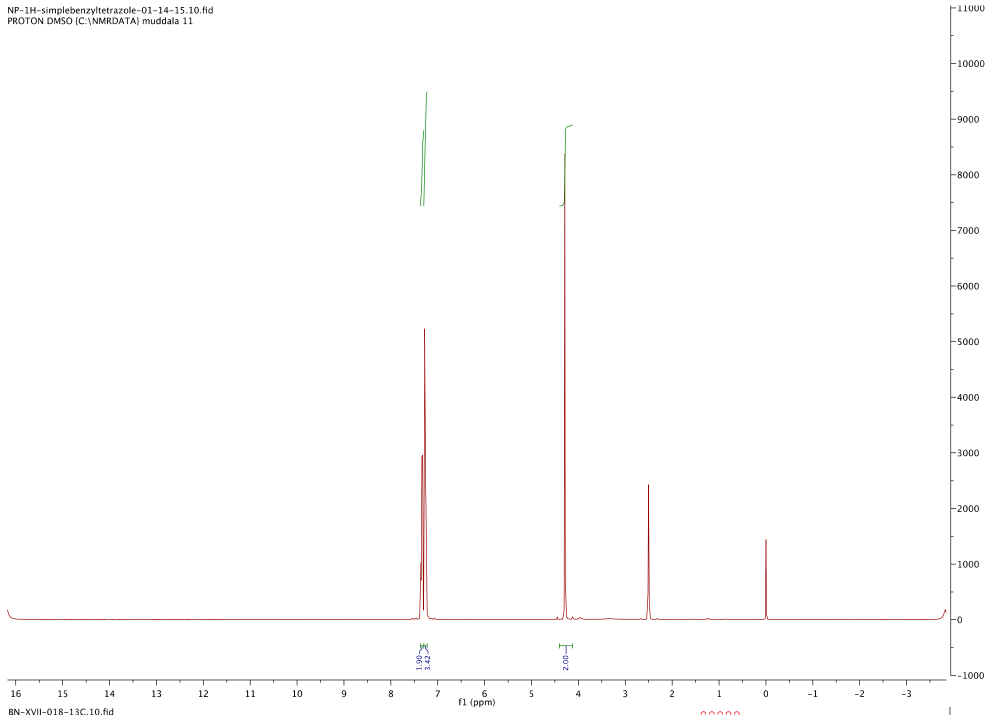

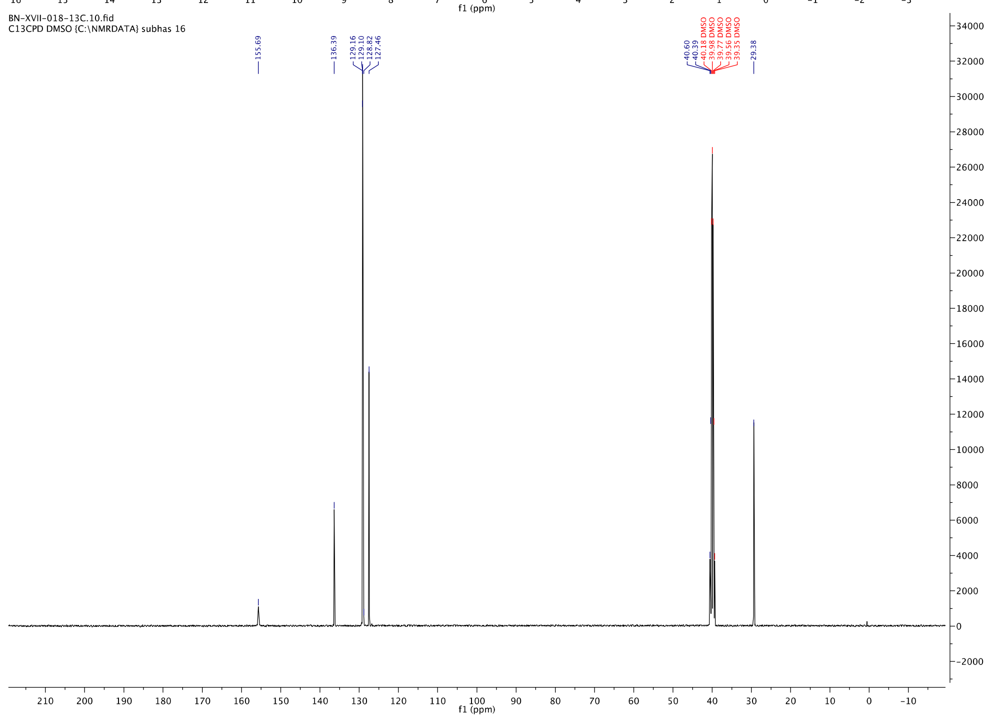

5-(4-Methylbenzyl)-1H-tetrazole (**2b**)

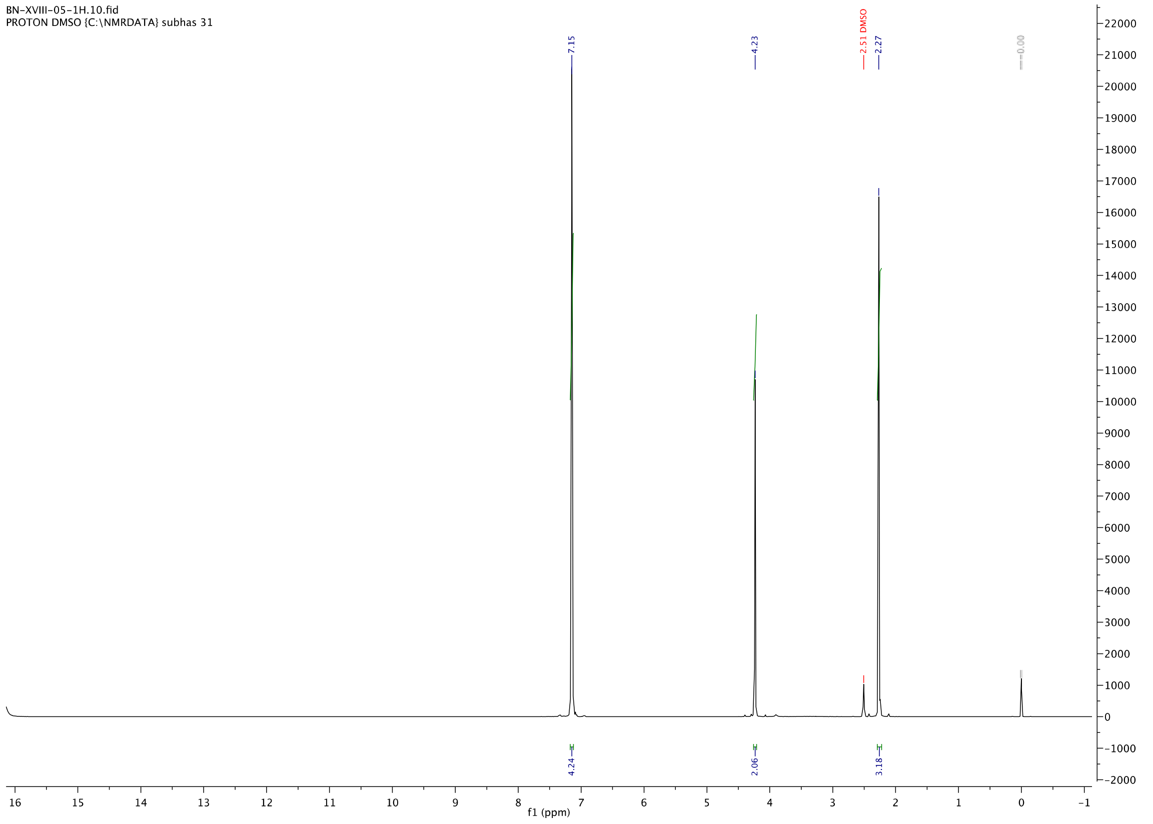

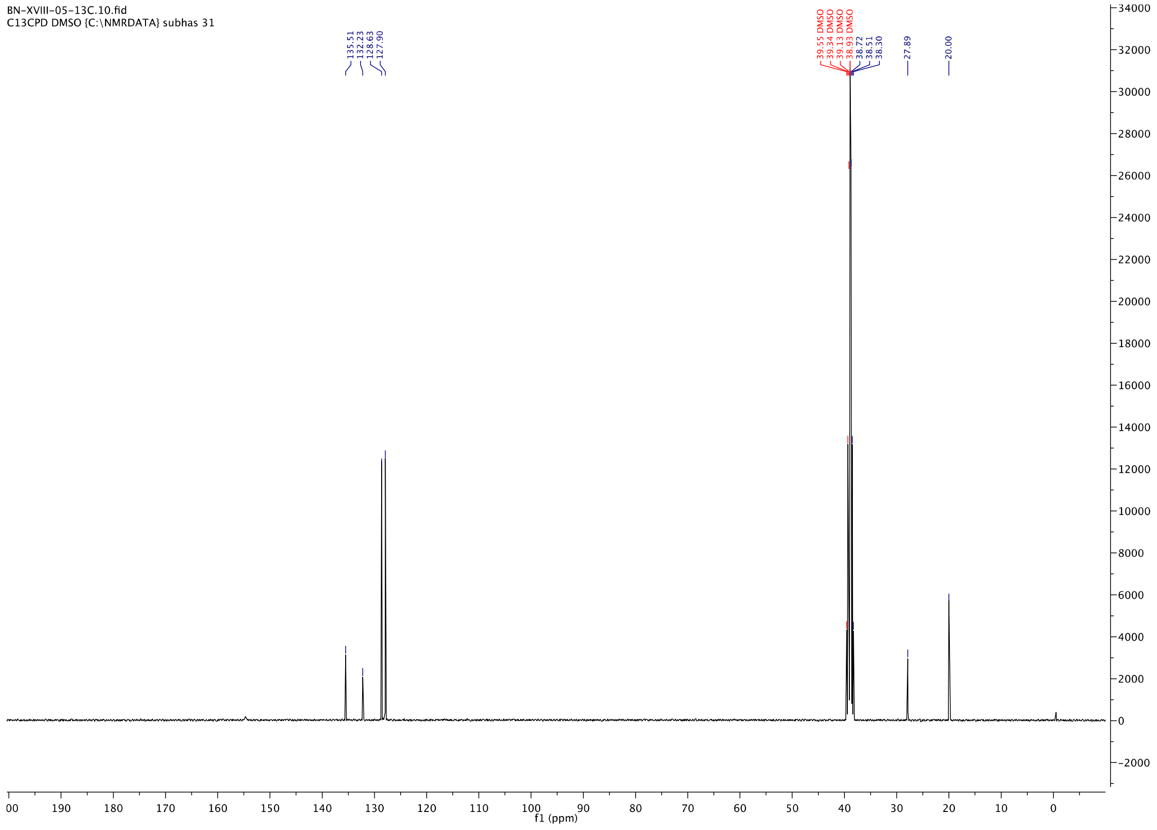

5-(4-Methoxybenzyl)-1H-tetrazole (**2c**)

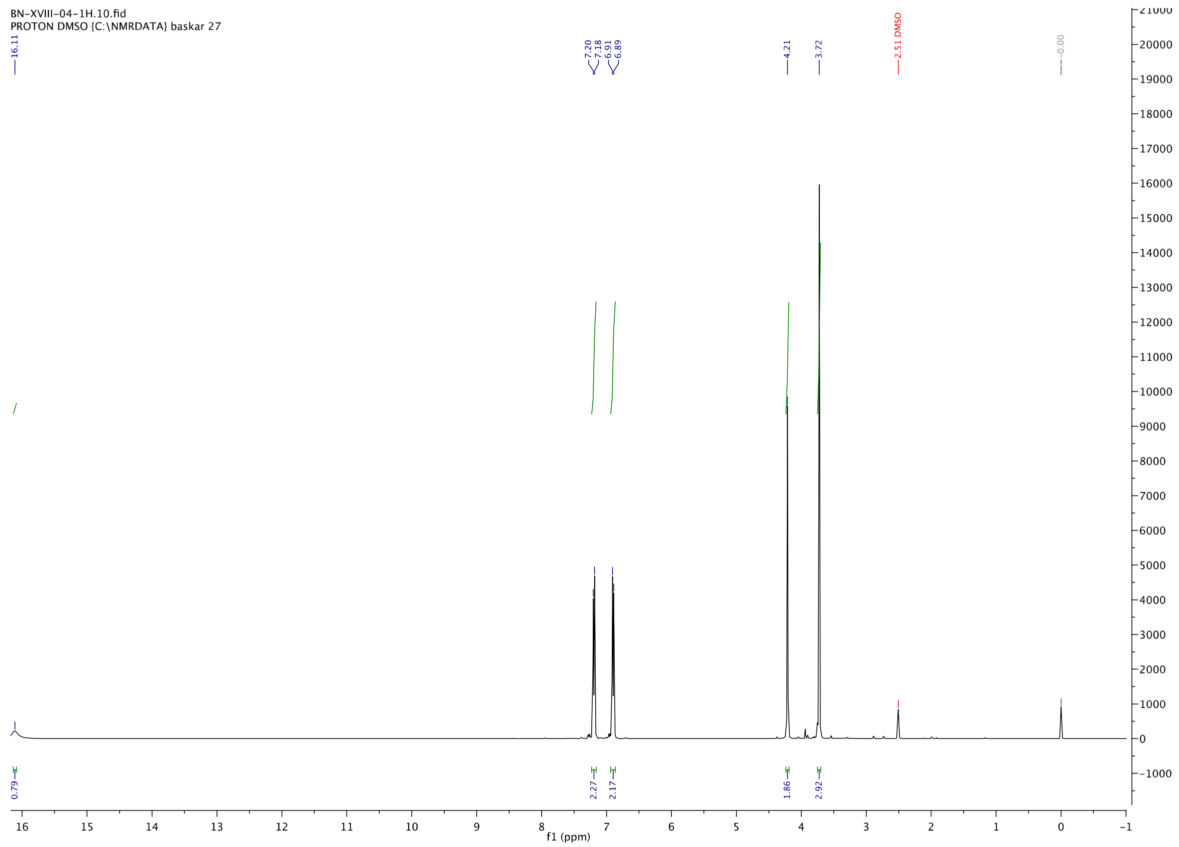

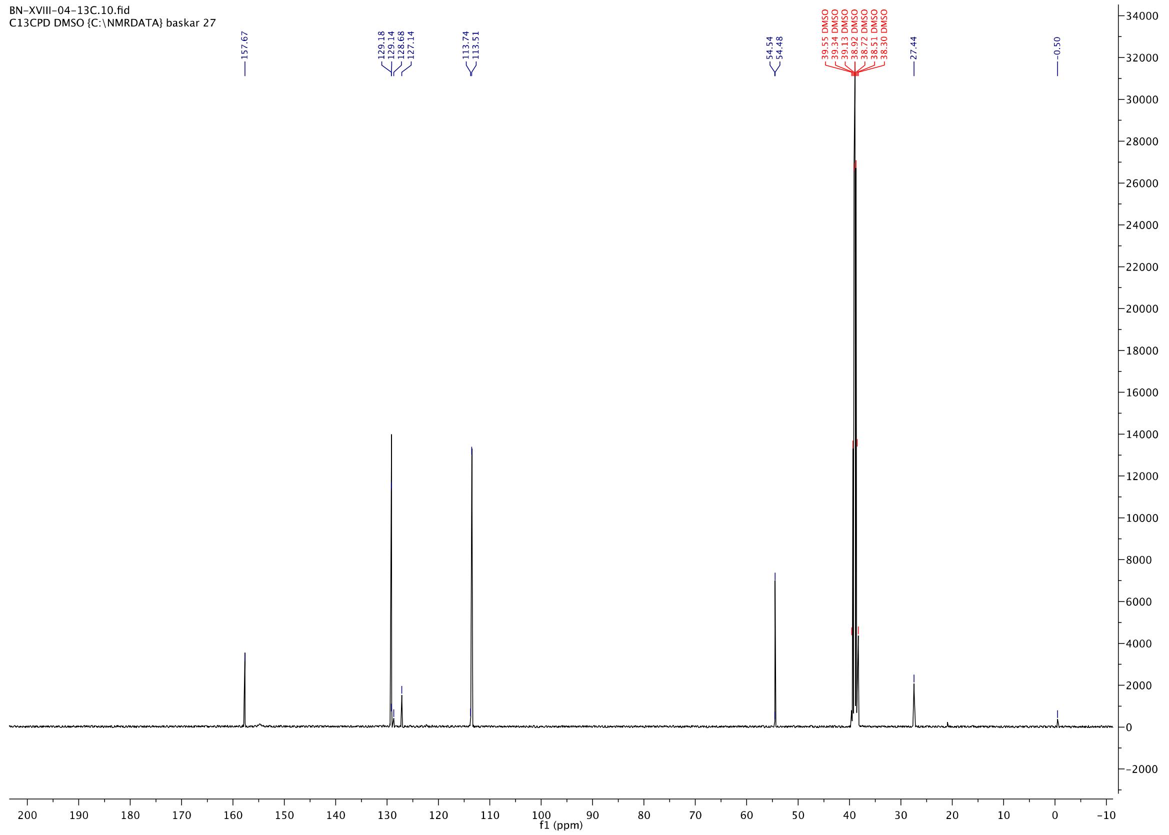

5-(4-Chlorobenzyl)-1H-tetrazole (**2d**)

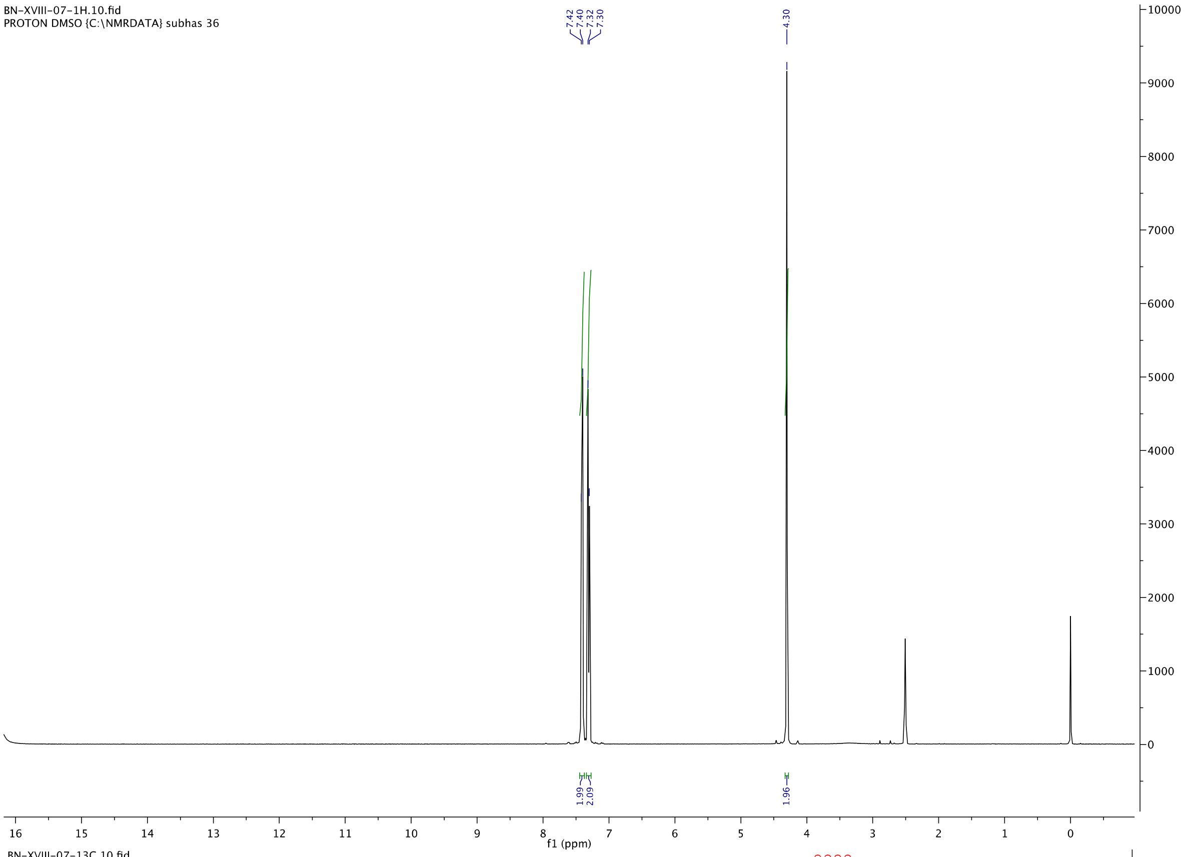

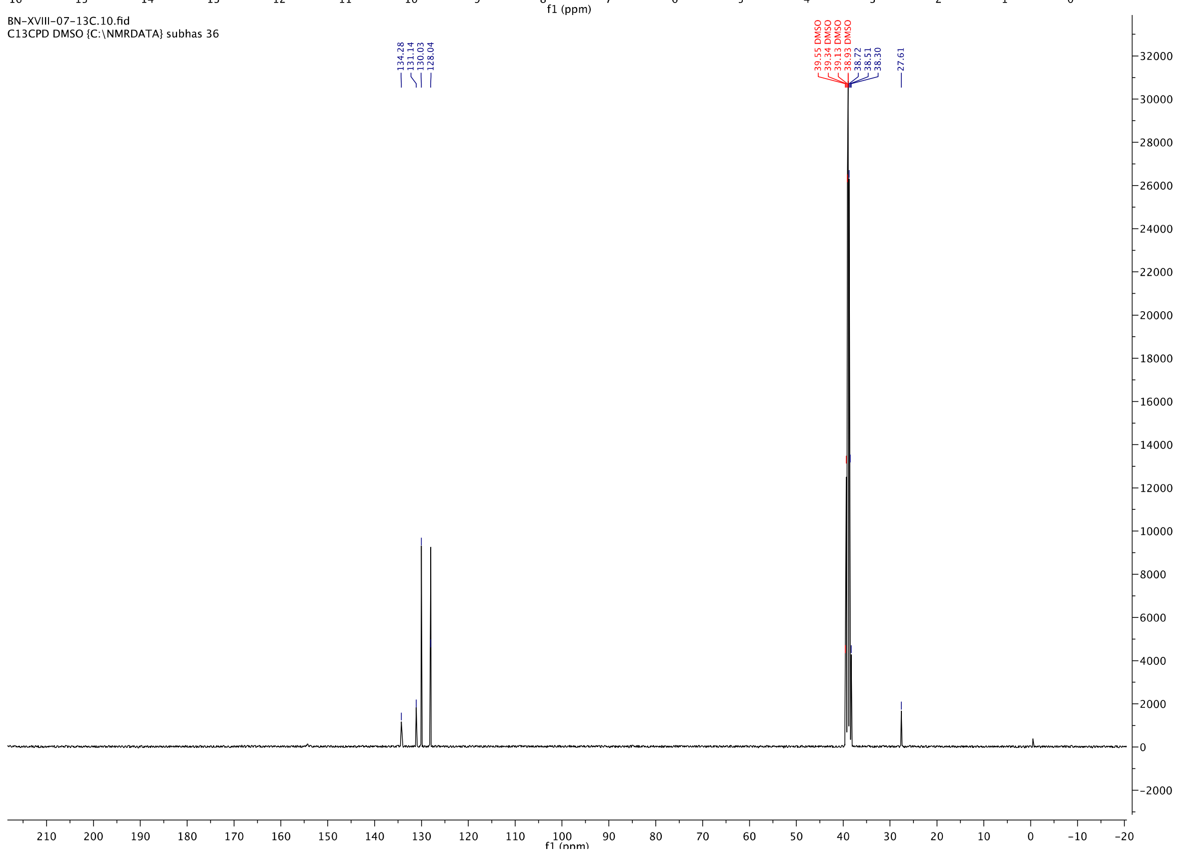

5-(4-Fluorobenzyl)-1H-tetrazole (**2e**)

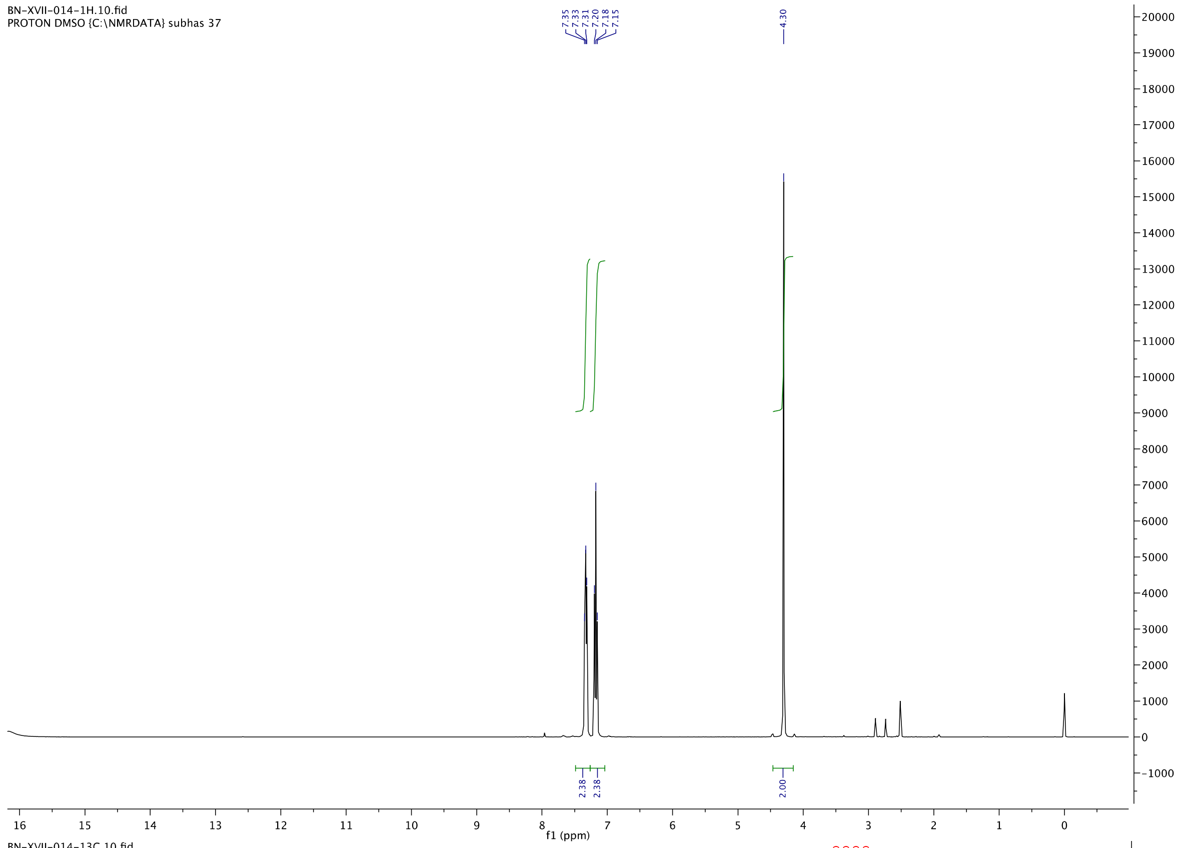

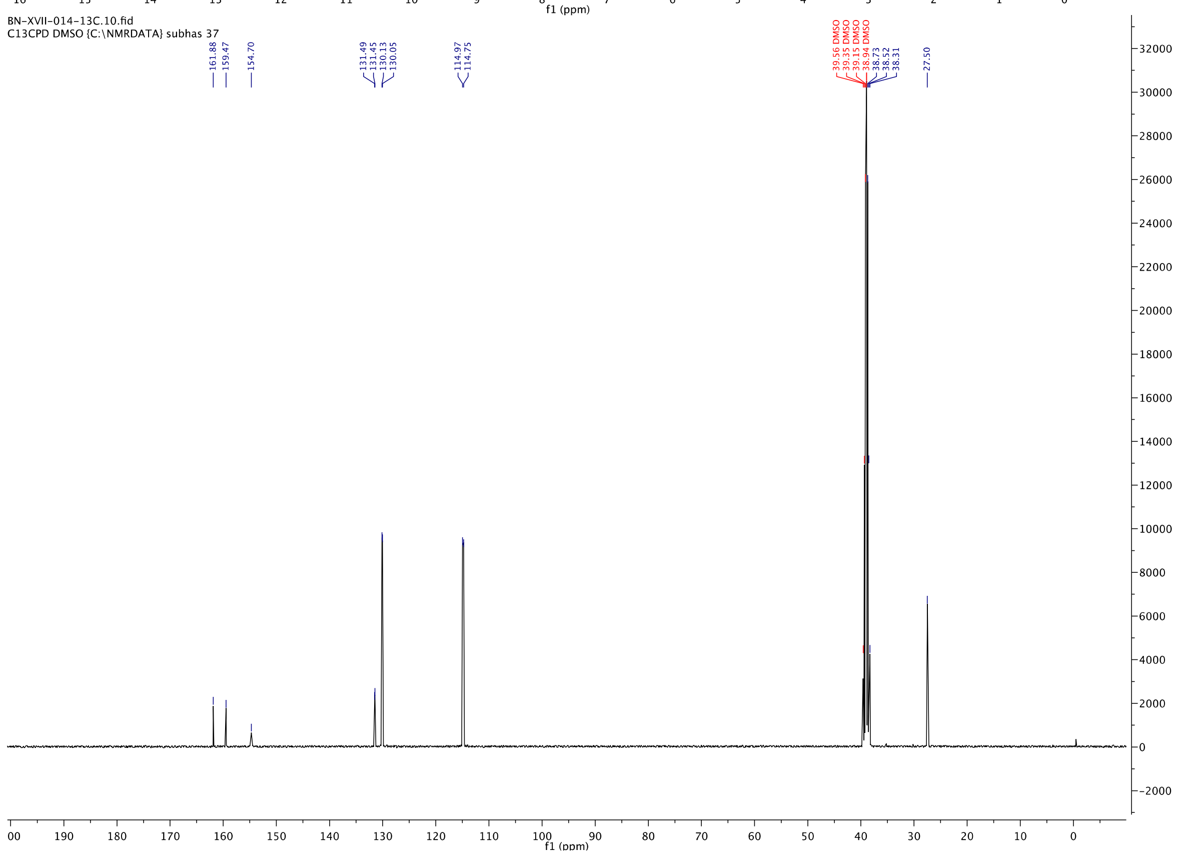

5-Phenyl-1H-tetrazole (**2f**)

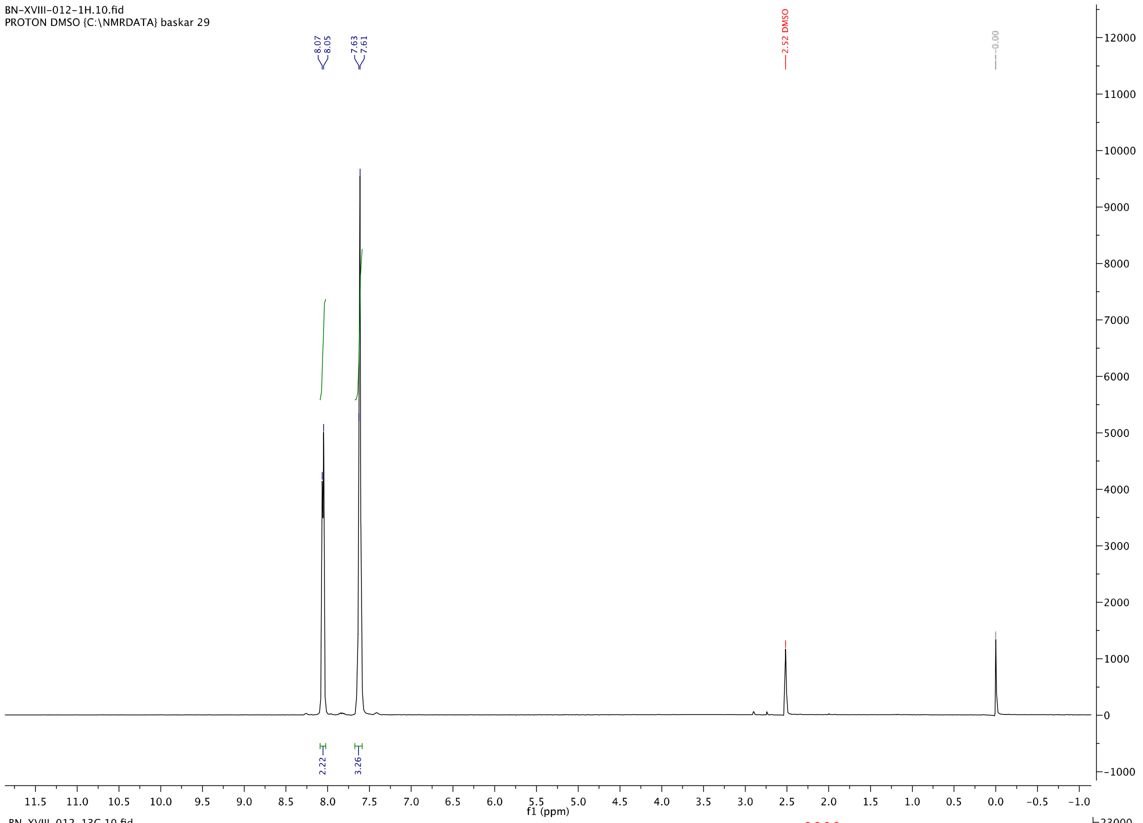

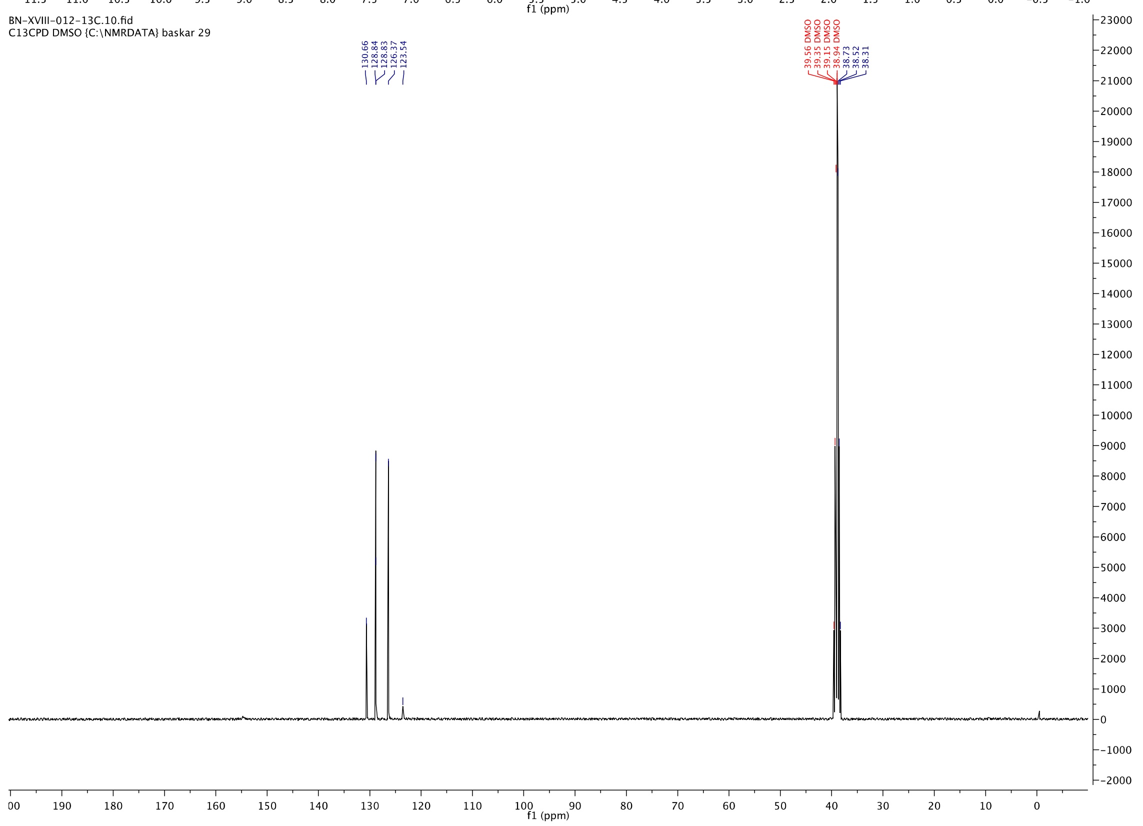

5-(4-Methylphenyl)-1H-tetrazole (**2g**)

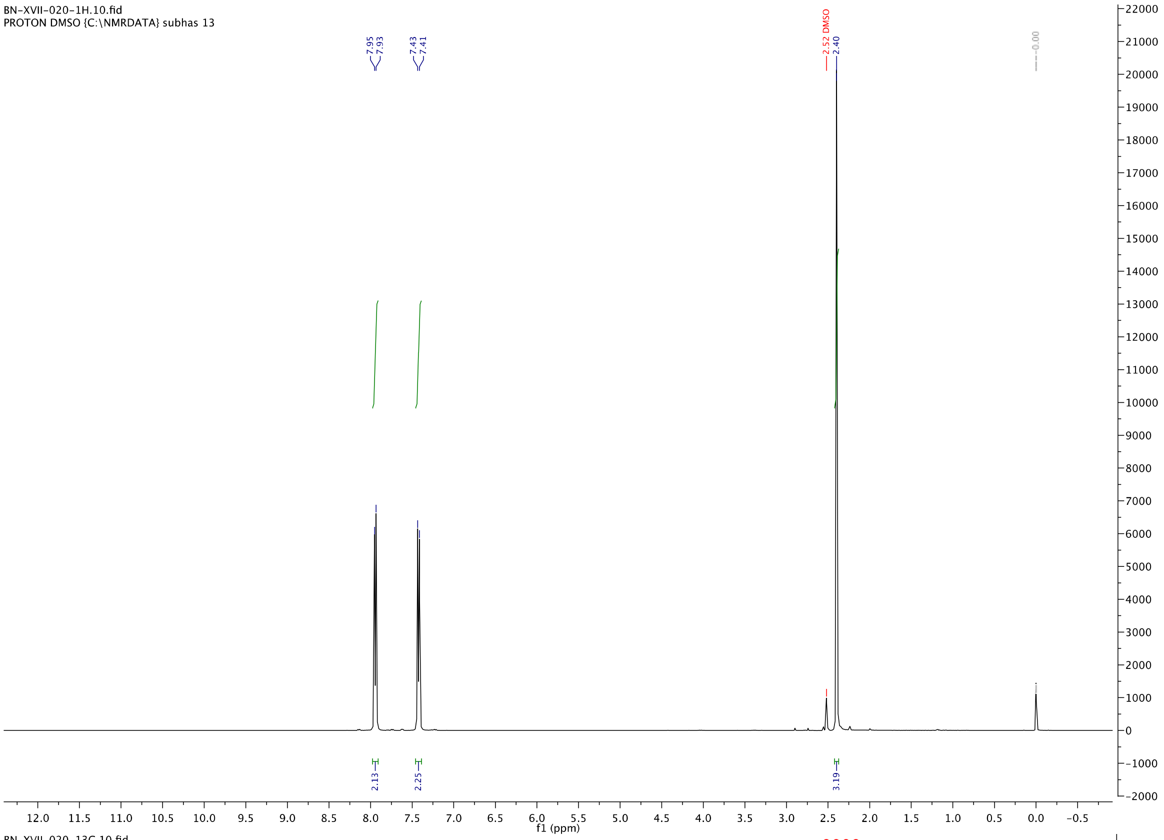

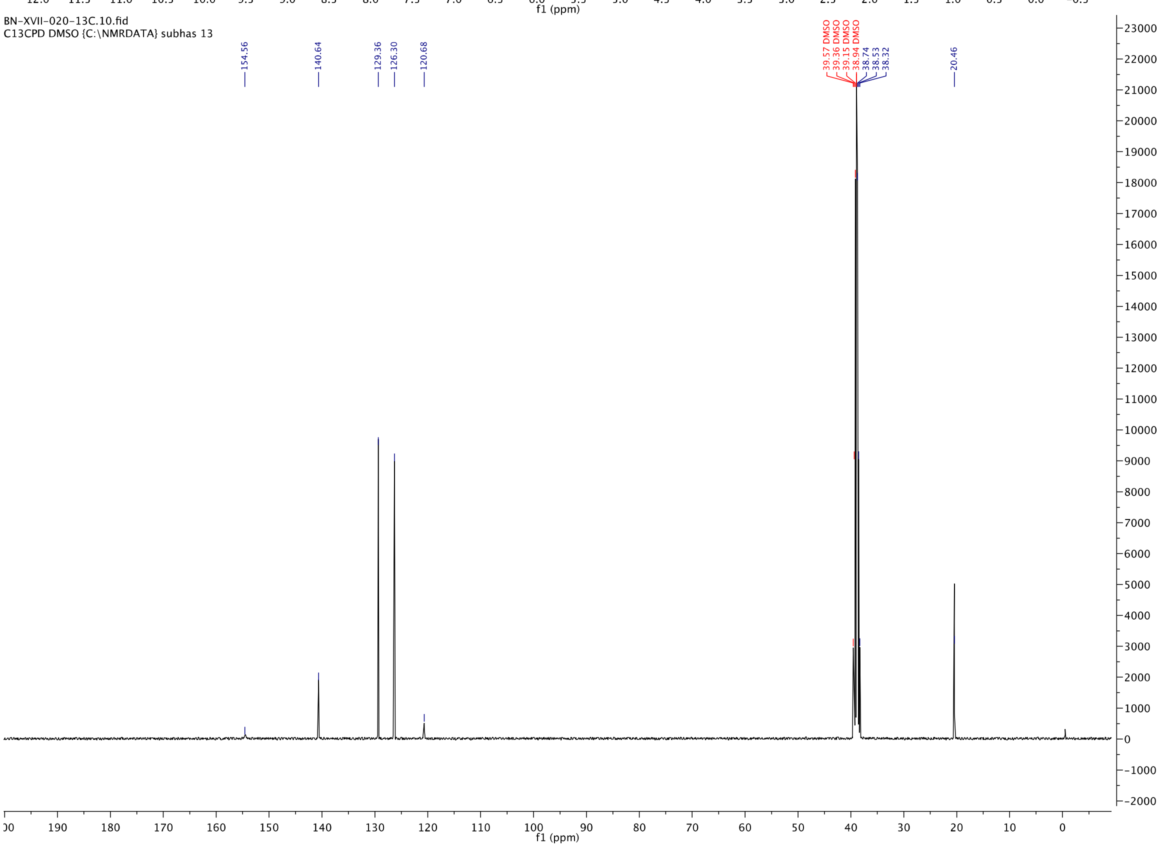

5-(2-Methylphenyl)-1H-tetrazole (**2h**)

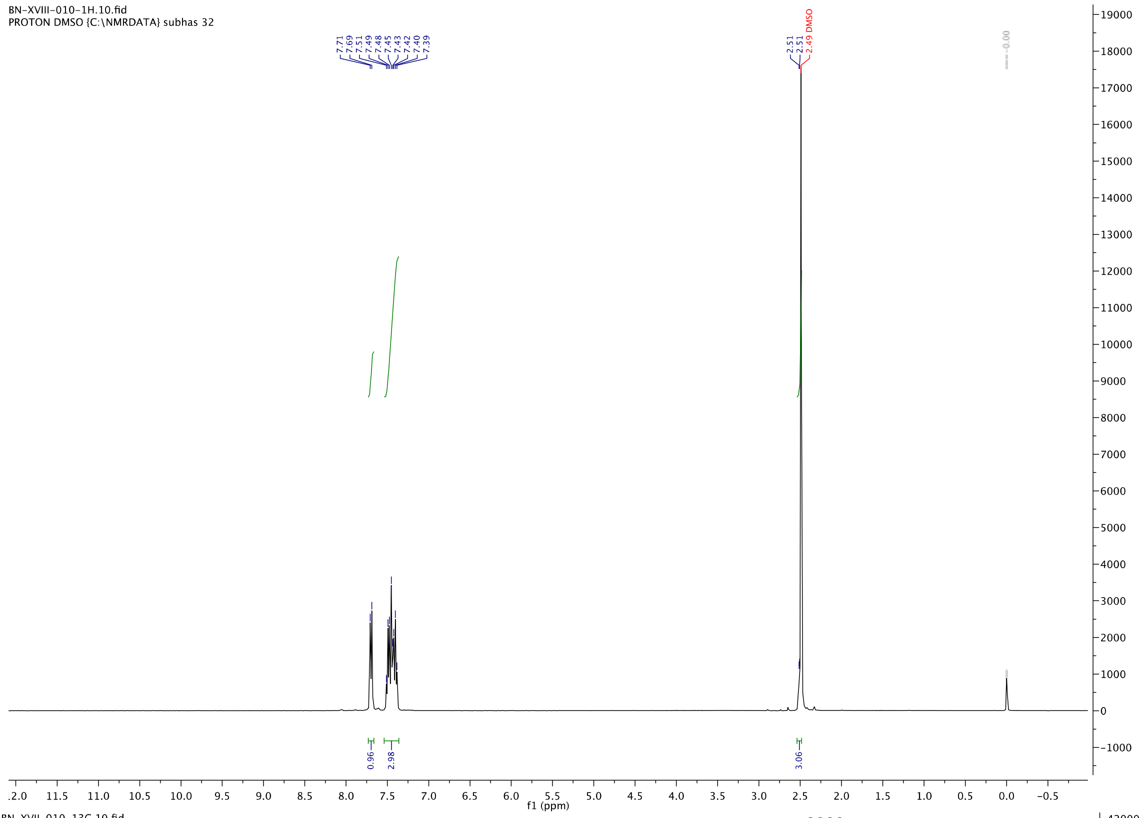

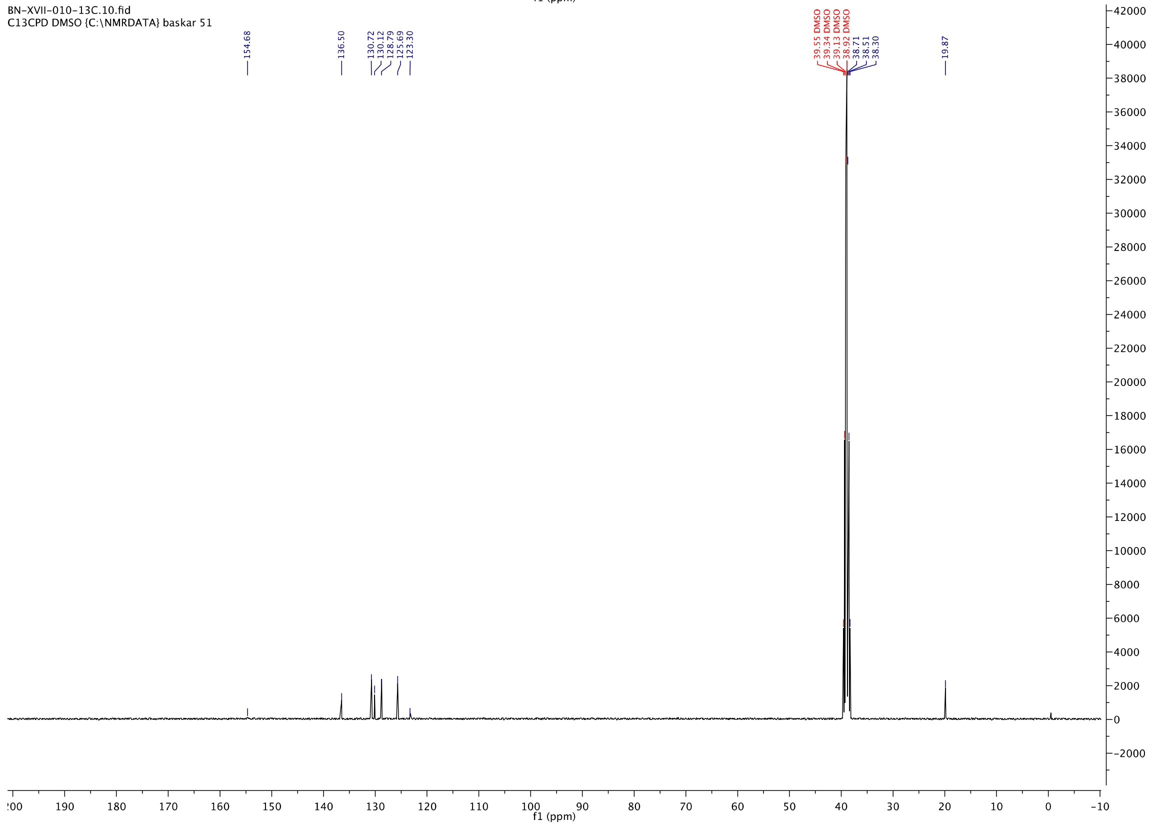

5-(4-Methoxyphenyl)-1H-tetrazole (**2i**)

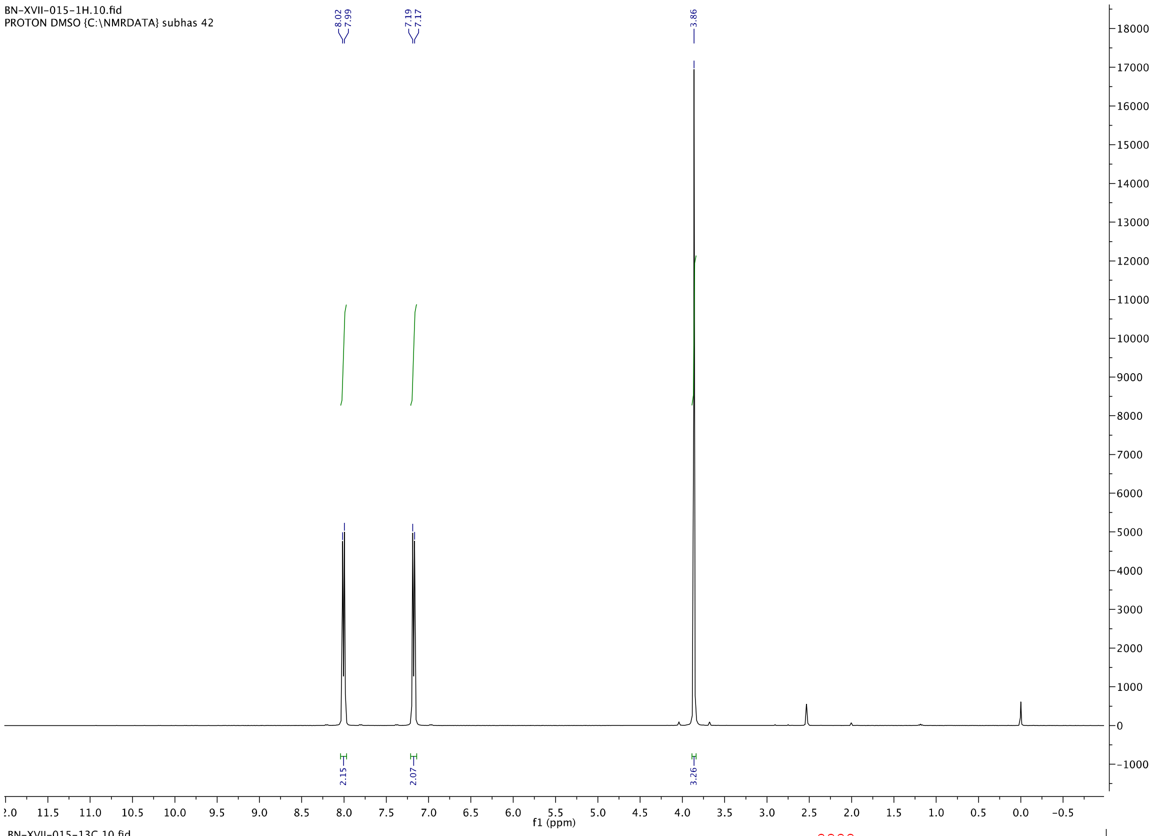

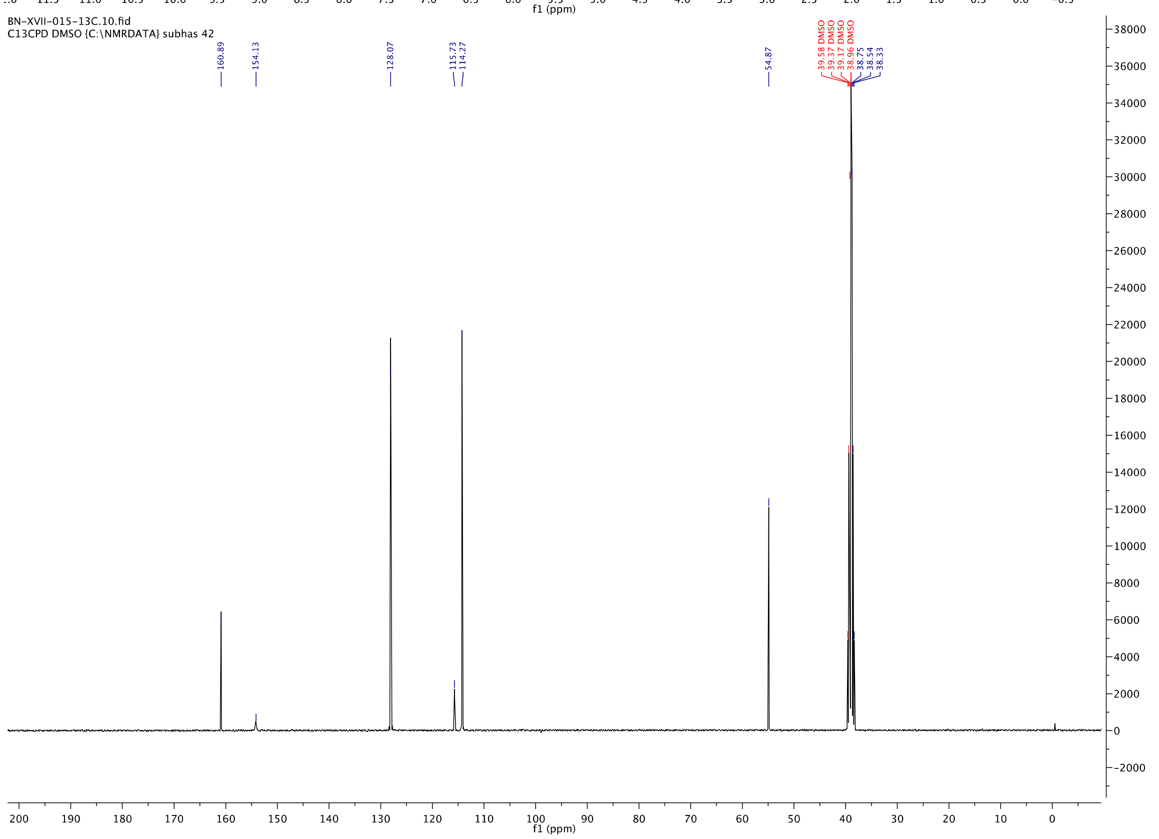

5-(4-Nitrophenyl)-1H-tetrazole (**2j**)

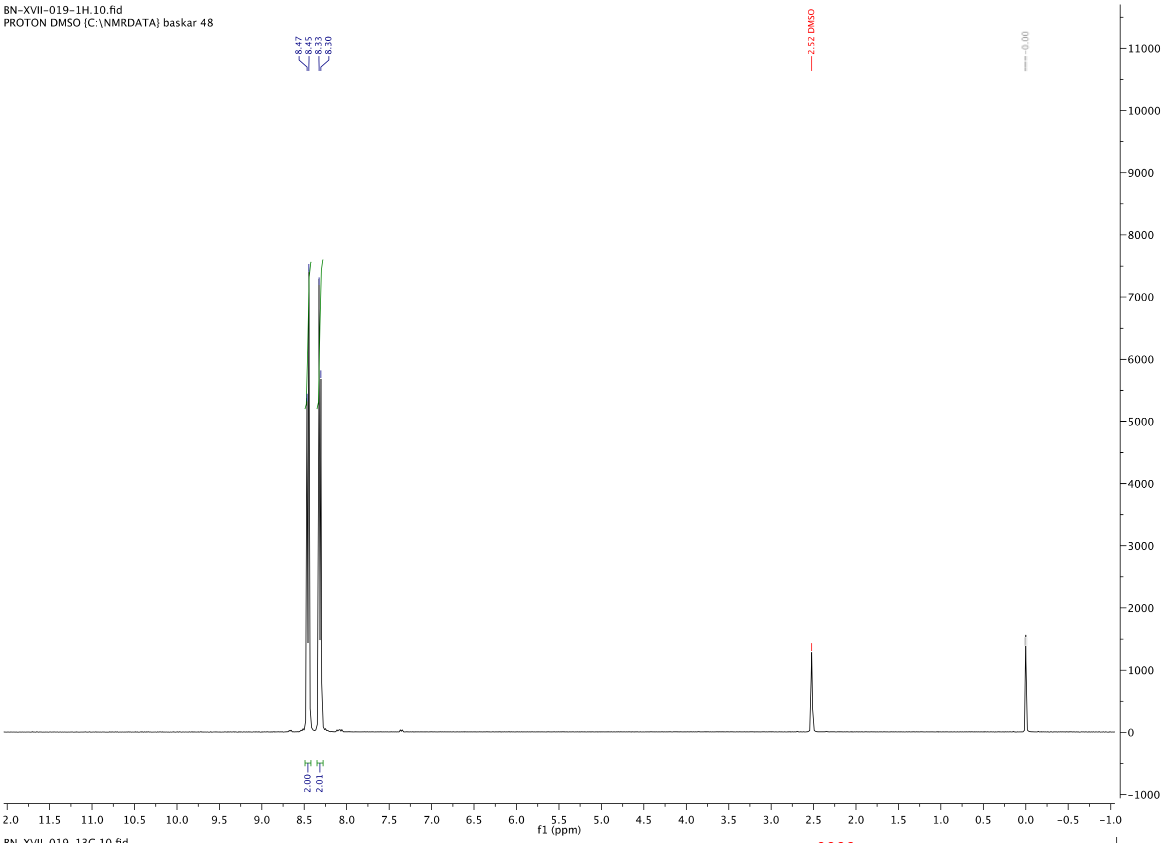

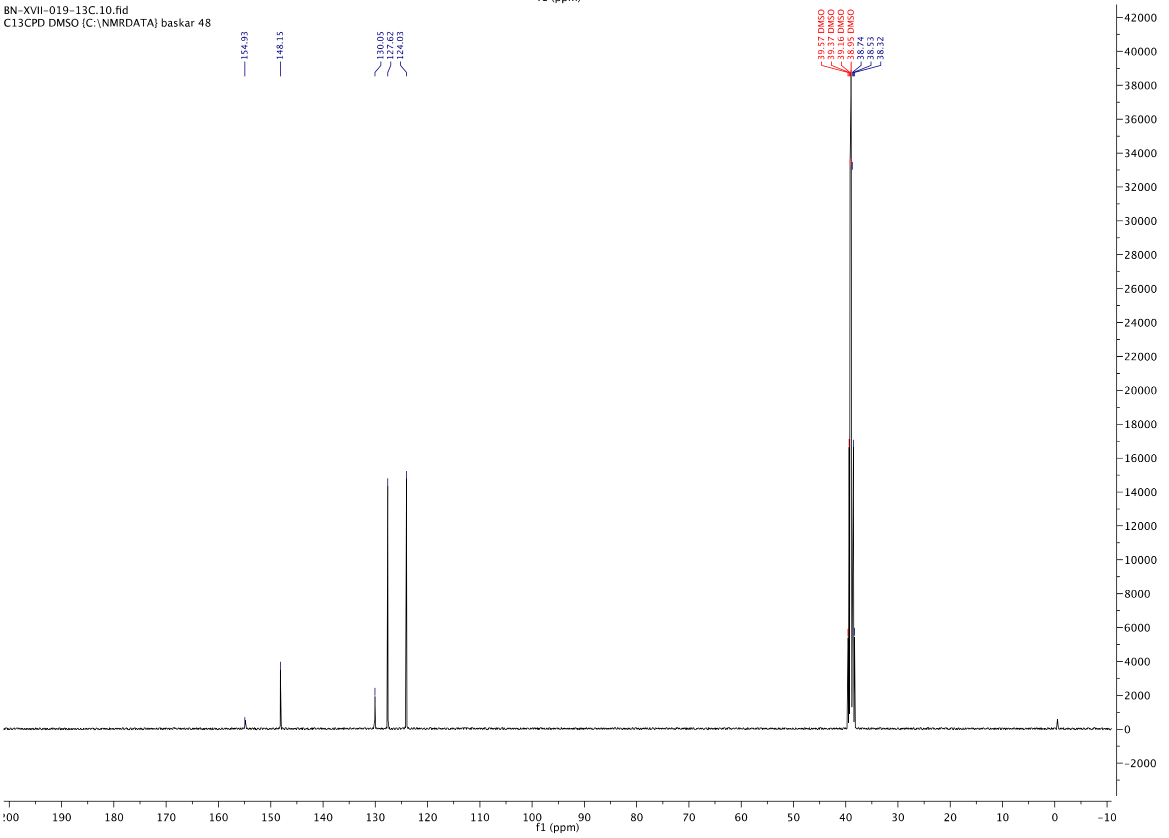

5-(3-Nitrophenyl)-1H-tetrazole (**2k**)

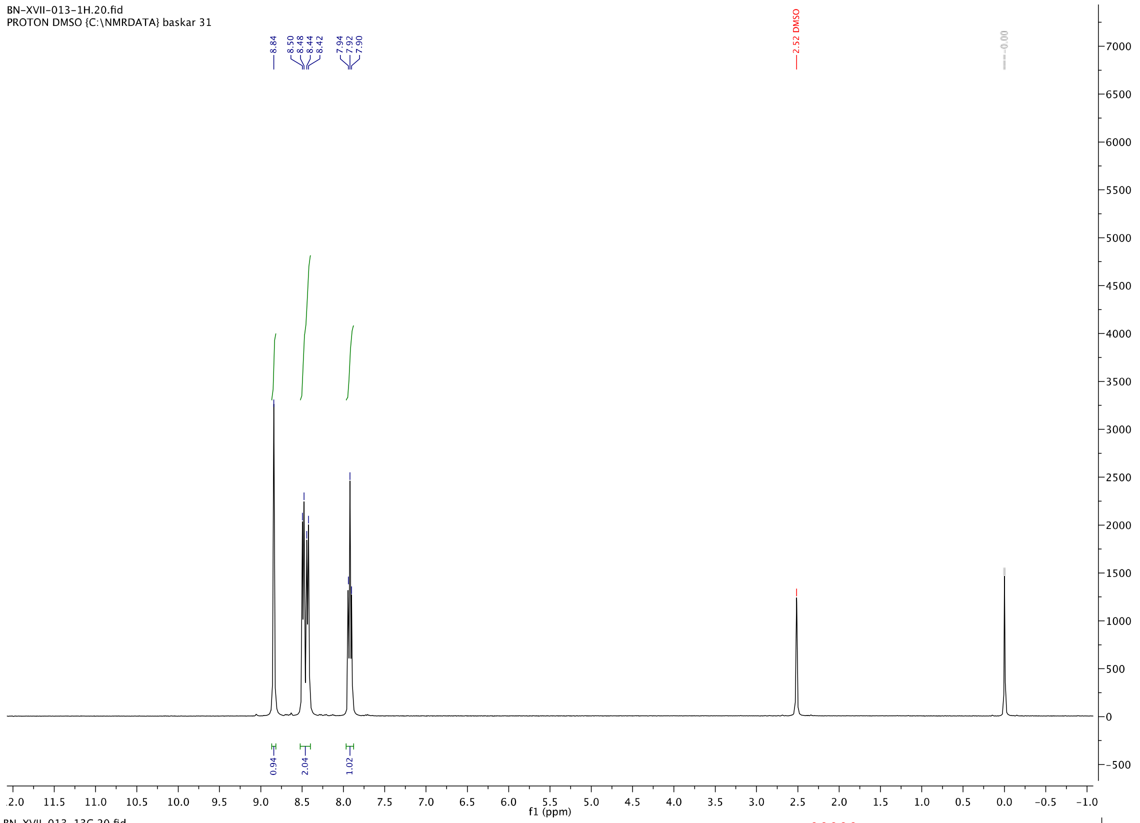

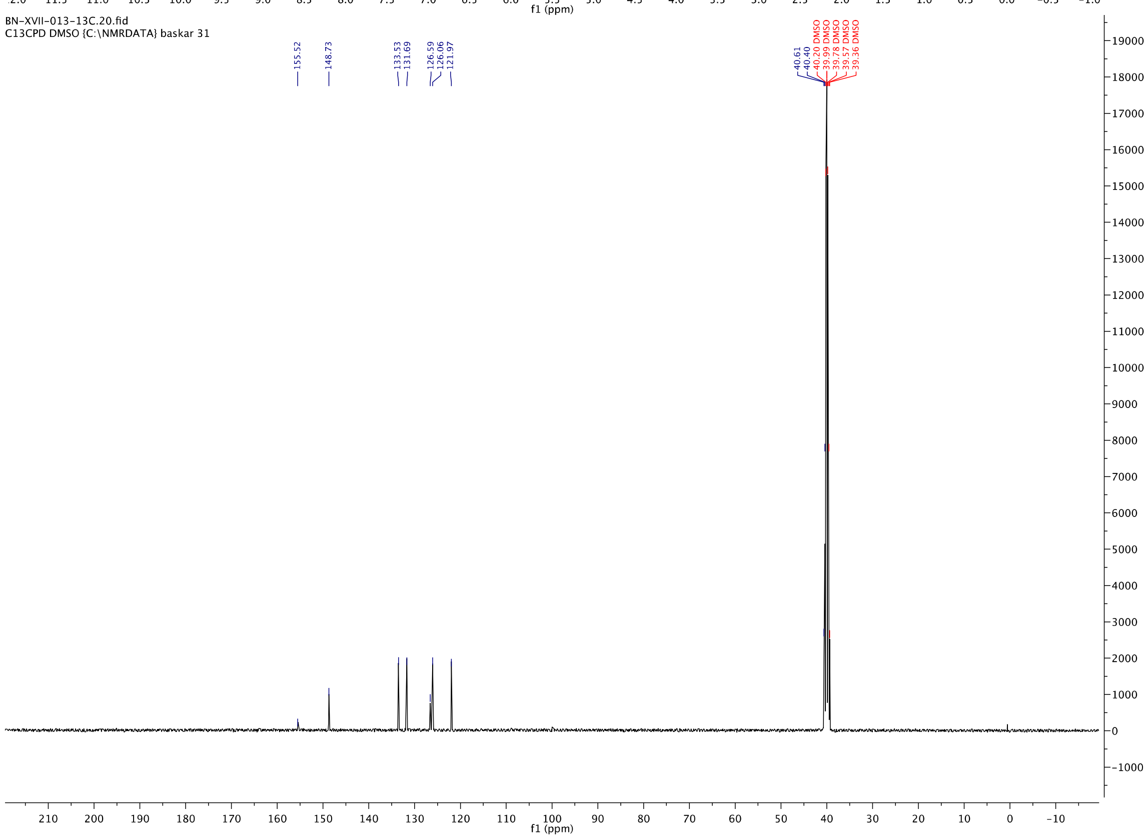

5-(4-Chlorophenyl)-1H-tetrazole (**2l**)

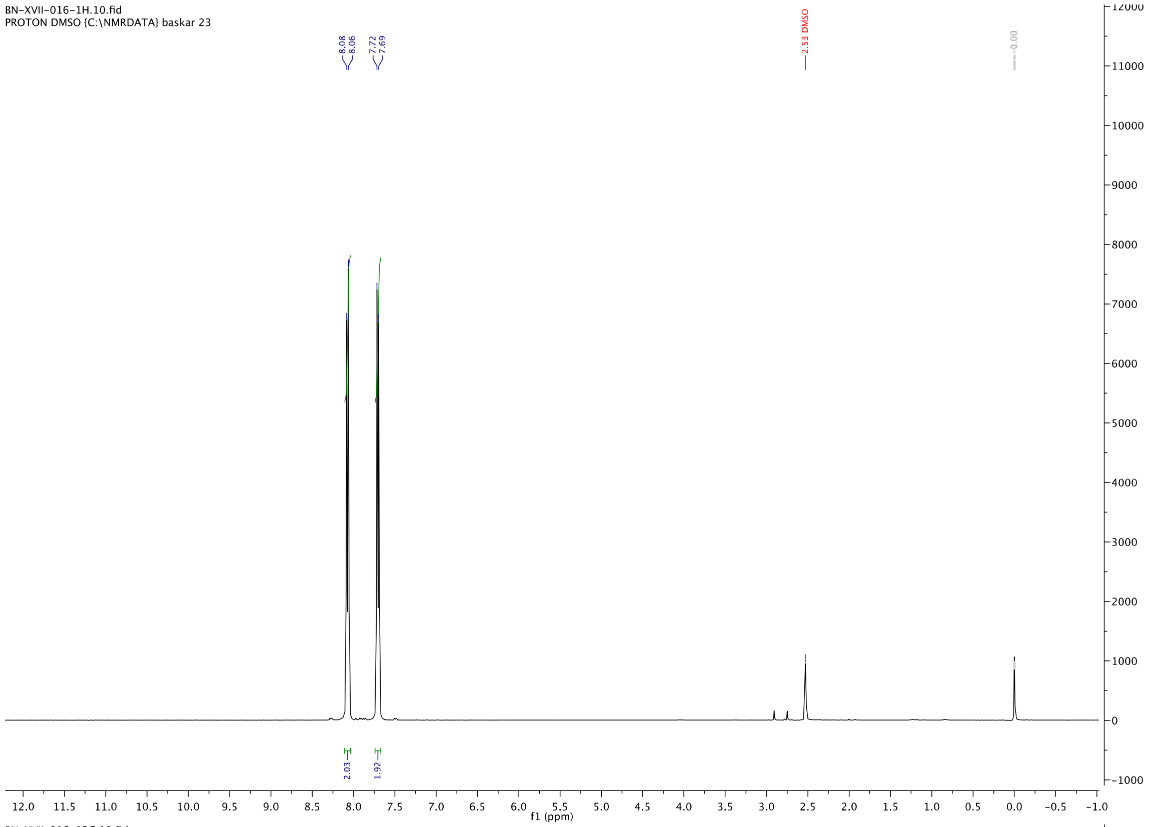

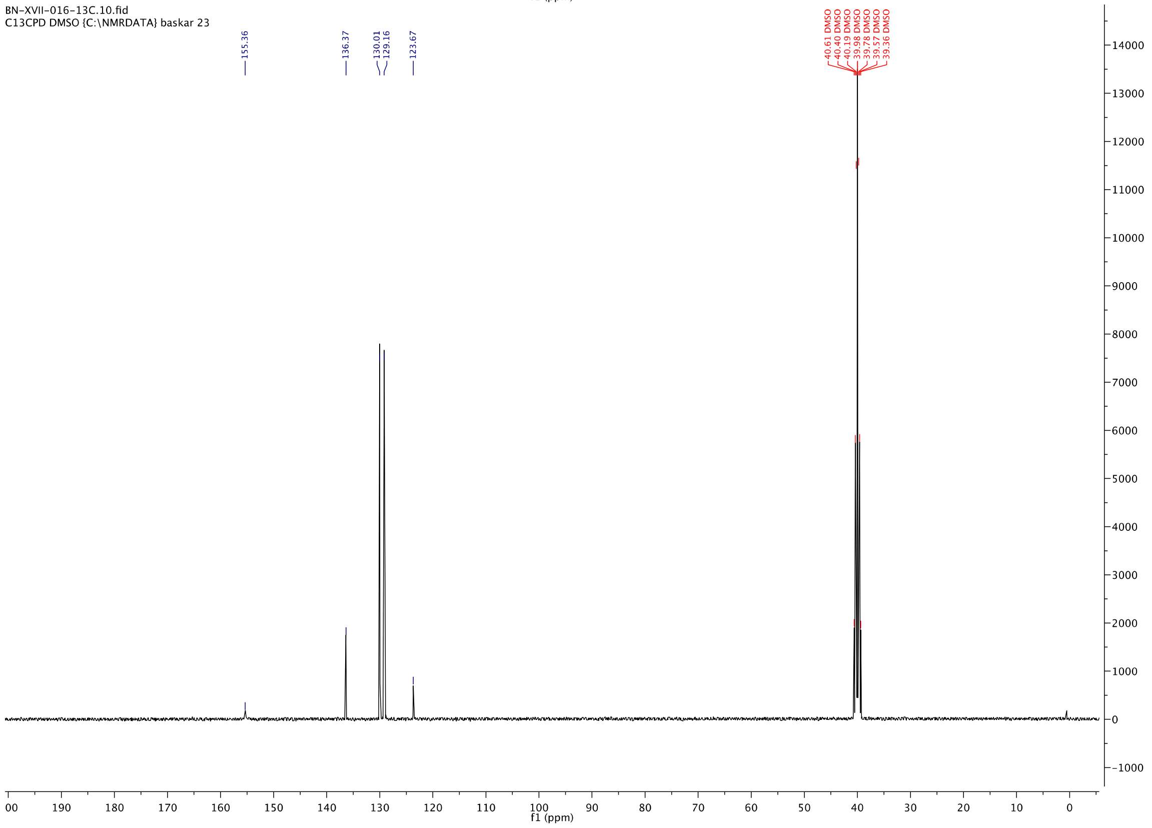

5-(4-Fluorophenyl)-1H-tetrazole (**2m**)

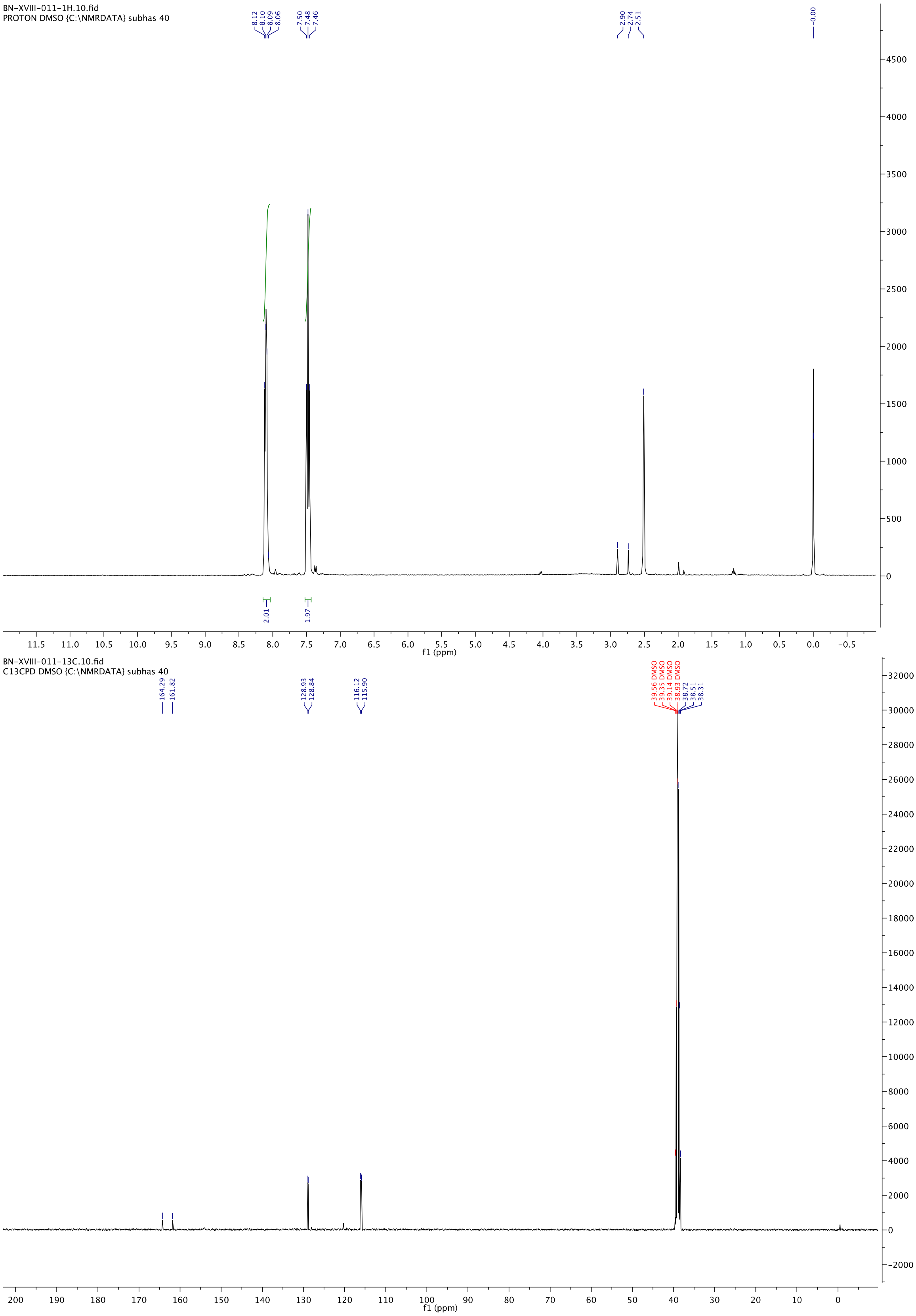

5-Diphenylmethyl-1H-tetrazole (**2n**)

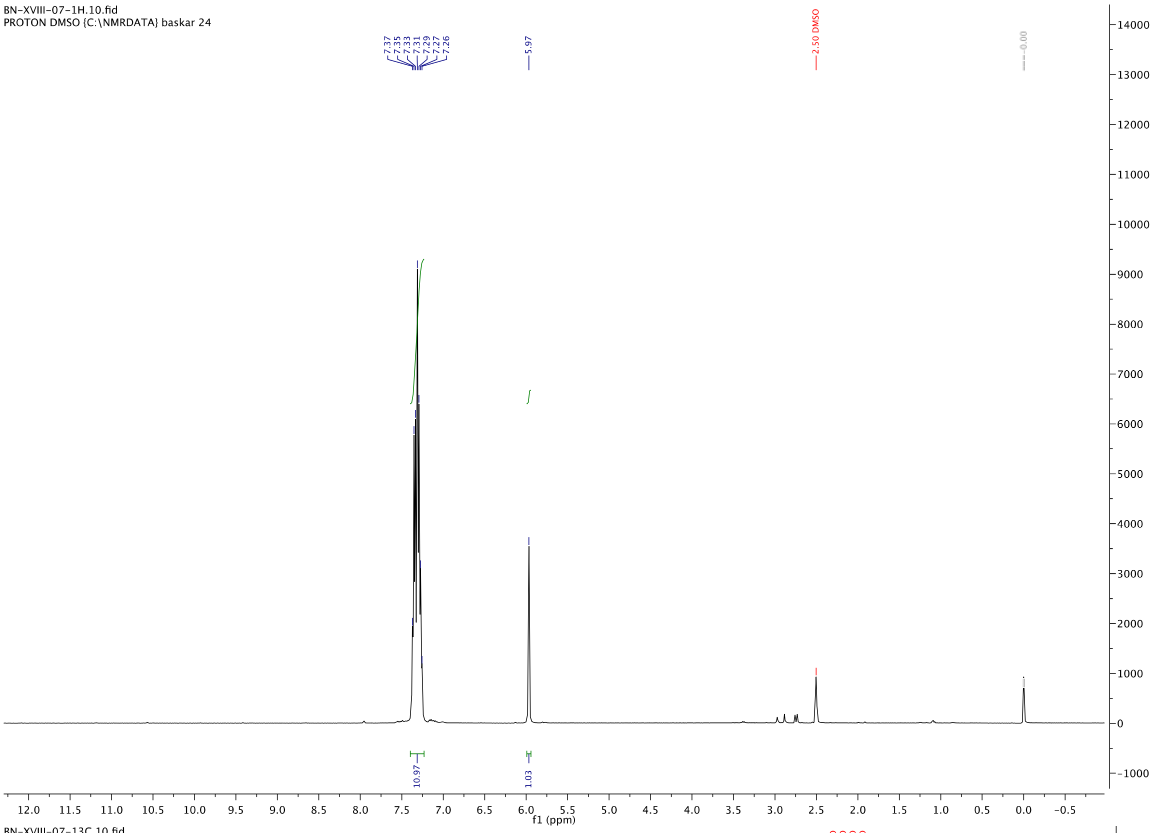

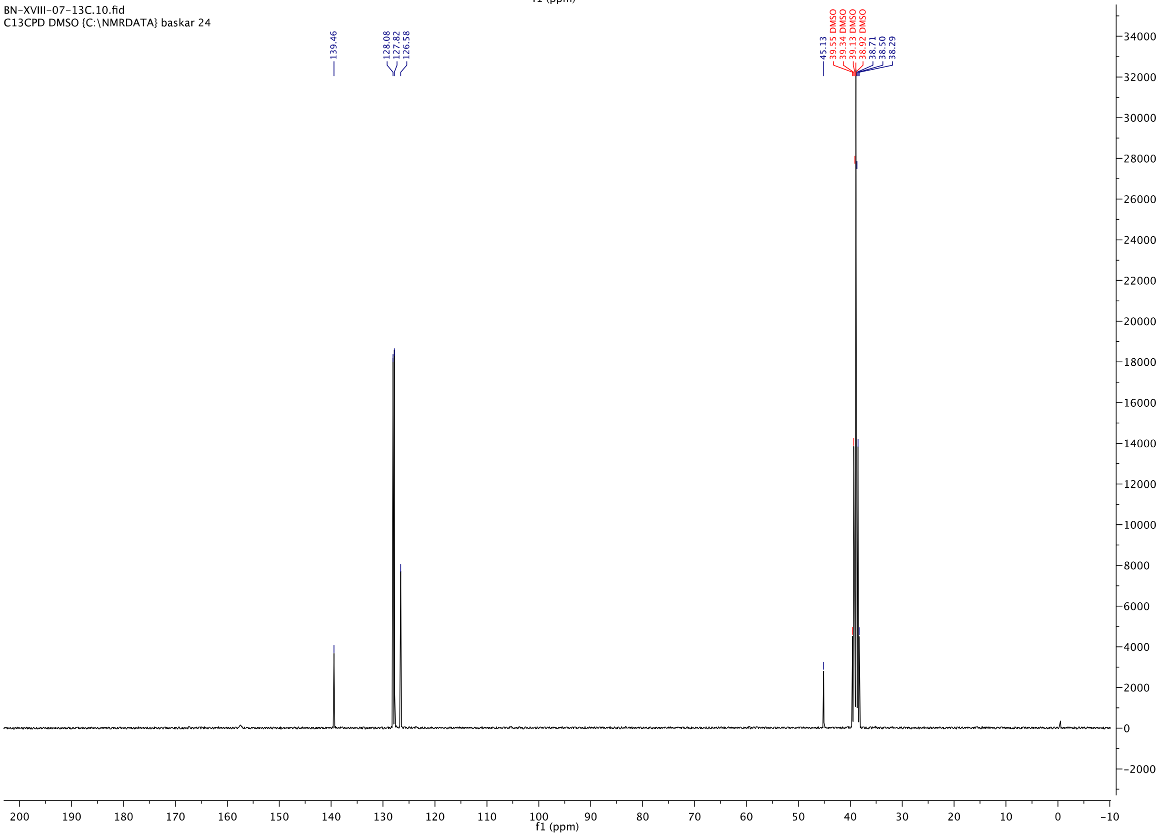

5-(4-Heptylphenyl)-1H-tetrazole (**2o**)

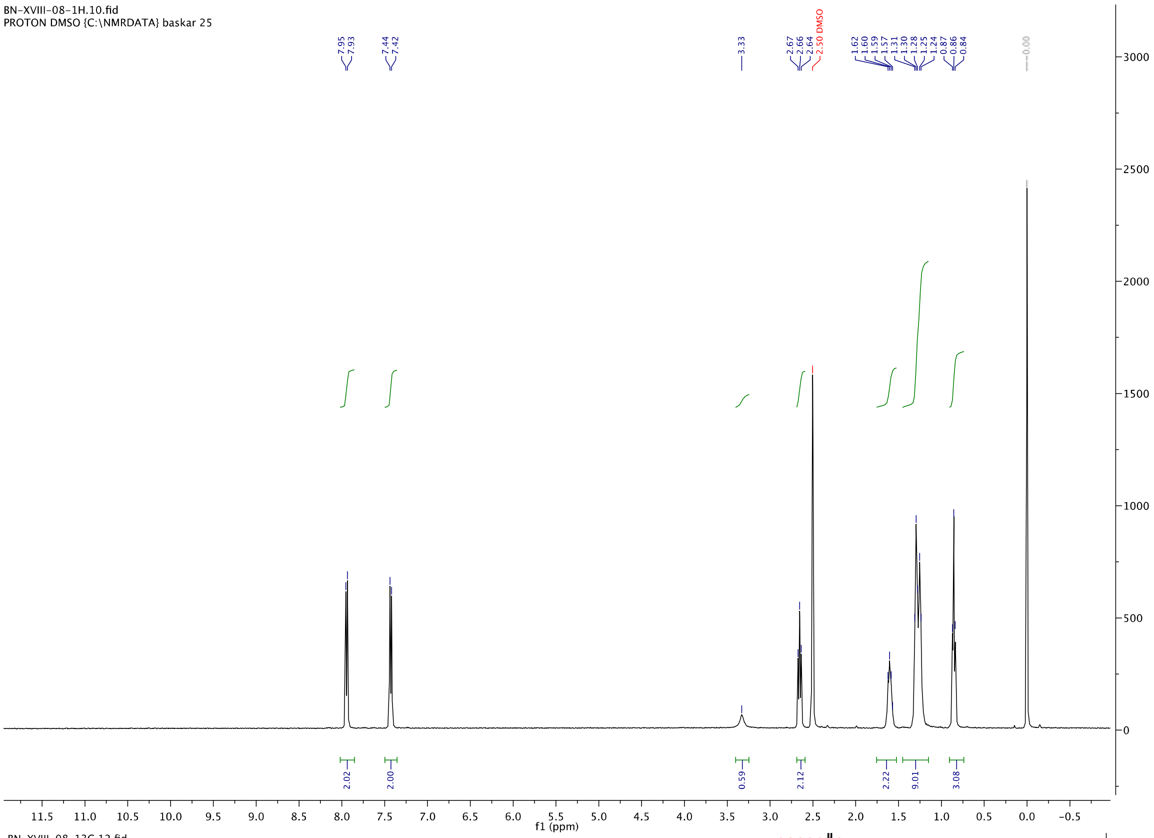

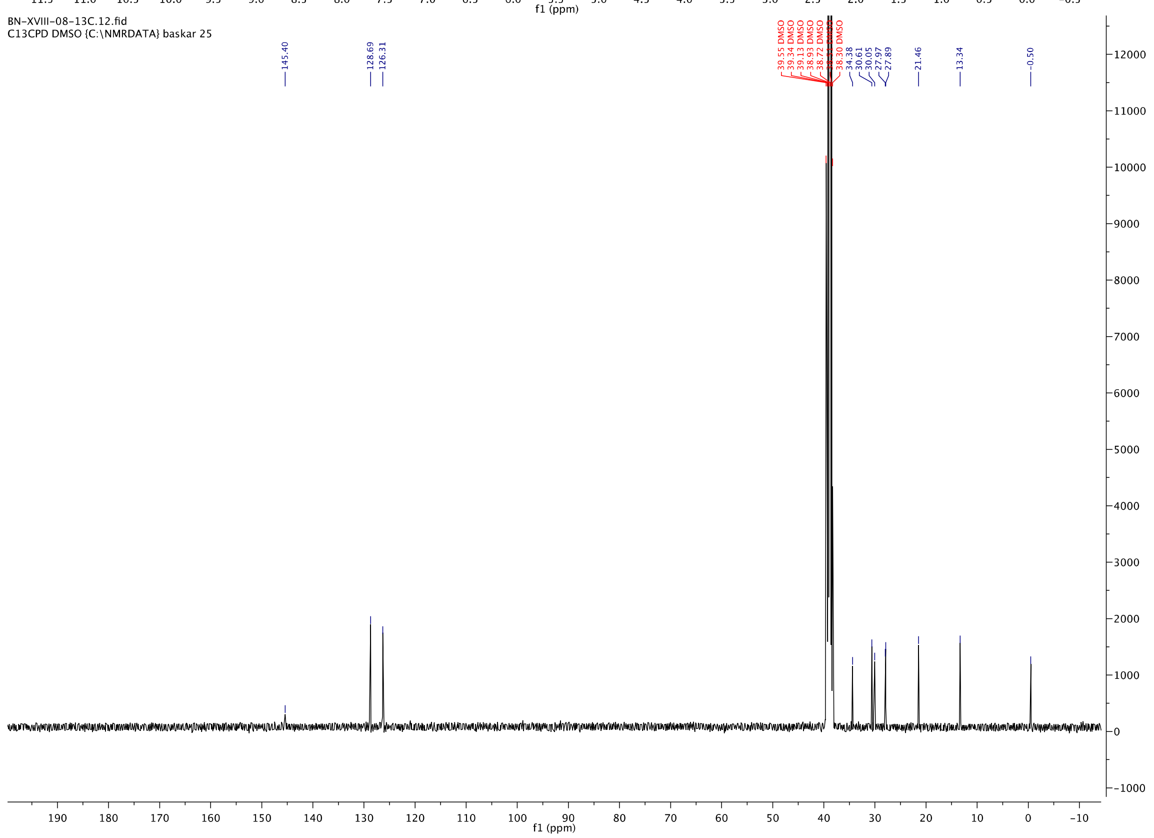

5-[4-(3-Butenyl)phenyl]-1H-tetrazole (**2p**)

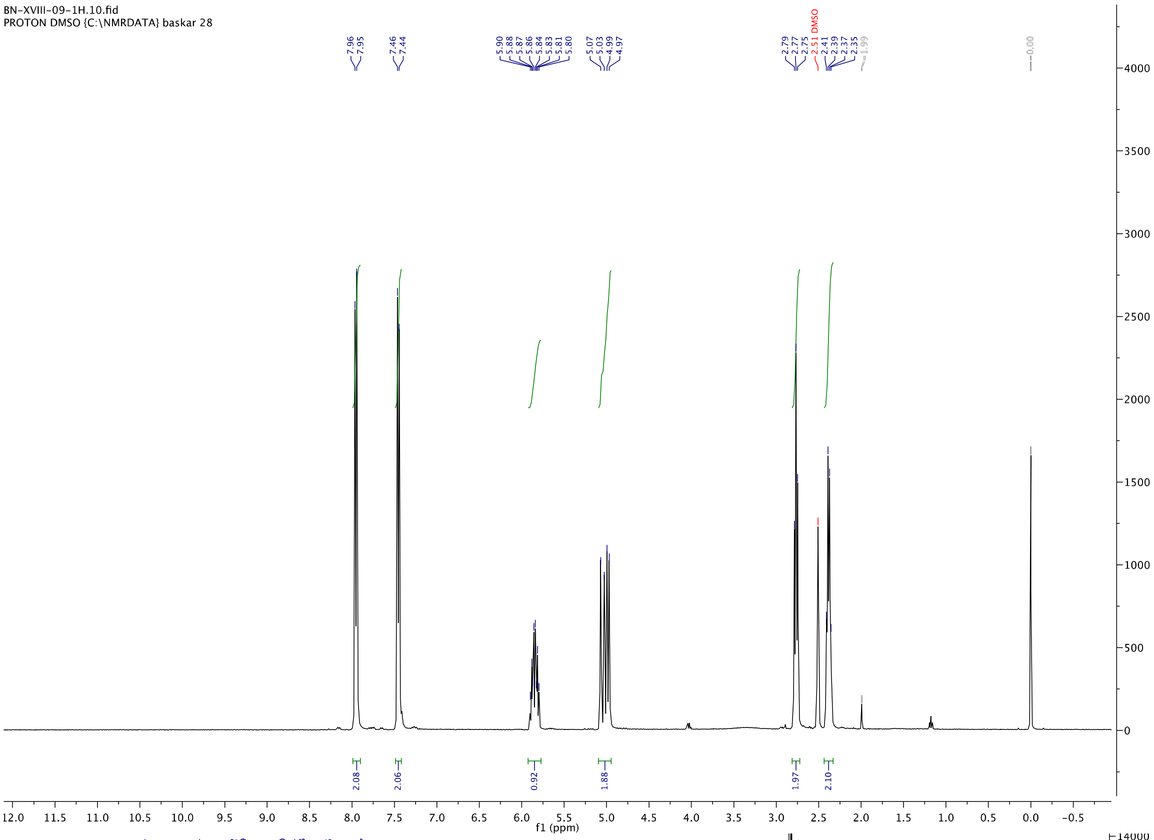

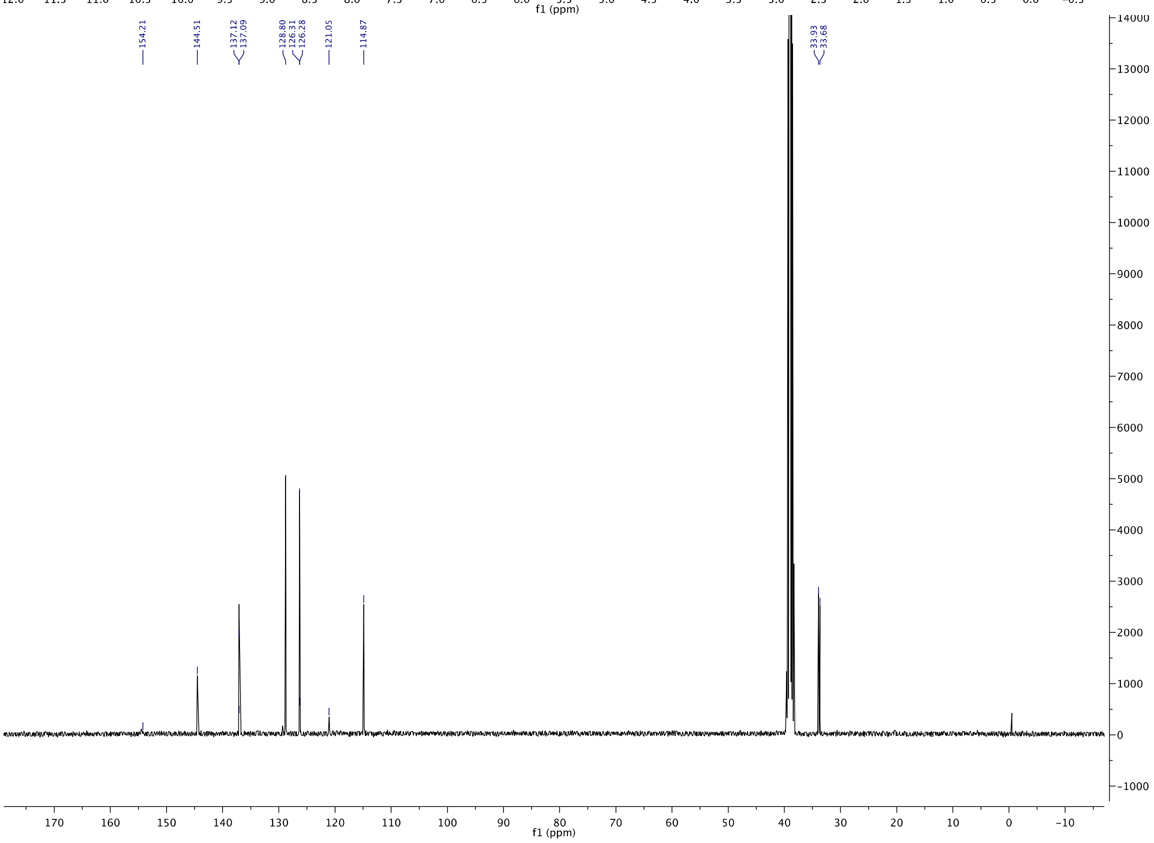

Supplement: Supplementary file 1 [file molecules-20-19881-s001.pdf]
